# Surveying Wearable Human Assistive Technology for Life and Safety Critical Applications: Standards, Challenges and Opportunities

**DOI:** 10.3390/s140509153

**Published:** 2014-05-23

**Authors:** Muhammad Mahtab Alam, Elyes Ben Hamida

**Affiliations:** Qatar Mobility Innovations Center (QMIC), Qatar Science and Technology Park (QSTP), PO Box 210531, Doha, Qatar; E-Mail: elyesb@qmic.com

**Keywords:** wearable technology, Wireless Body Area Networks (WBANs), critical and safety applications, IEEE 802.15.6 standard, intra-WBANs, inter-WBANs

## Abstract

In this survey a new application paradigm life and safety for critical operations and missions using wearable Wireless Body Area Networks (WBANs) technology is introduced. This paradigm has a vast scope of applications, including disaster management, worker safety in harsh environments such as roadside and building workers, mobile health monitoring, ambient assisted living and many more. It is often the case that during the critical operations and the target conditions, the existing infrastructure is either absent, damaged or overcrowded. In this context, it is envisioned that WBANs will enable the quick deployment of *ad-hoc*/on-the-fly communication networks to help save many lives and ensuring people's safety. However, to understand the applications more deeply and their specific characteristics and requirements, this survey presents a comprehensive study on the applications scenarios, their context and specific requirements. It explores details of the key enabling standards, existing state-of-the-art research studies, and projects to understand their limitations before realizing aforementioned applications. Application-specific challenges and issues are discussed comprehensively from various perspectives and future research and development directions are highlighted as an inspiration for new innovative solutions. To conclude, this survey opens up a good opportunity for companies and research centers to investigate old but still new problems, in the realm of wearable technologies, which are increasingly evolving and getting more and more attention recently.

## 1. Introduction

The increase in average lifespan and the pressure on health budgets in many developed countries have resulted to be important catalysts for the development of innovative and cost-effective health care solutions. Human assistive and wearable technologies such as Wireless Body Area Networks (WBANs) are emerging as an important part of the daily life for ambient assistive living. As an example it was recently reported that the UK's National Health Service (NHS) could save up to seven billions pounds per year by using innovative technologies to deliver quality healthcare to the chronically ill with fewer hospital visits and admissions [1].

In this regards the recent Consumer Electronics Show (CES'14 [2]) was a great showcase for wearable technologies. A number of products from companies such as Vuzix, Samsung, LG, Sony, Razer, *etc*., were introduced for applications such as medicine and fitness, entertainment, augmented reality, and many more. It is predicted that wearable technologies could be a key booster for the consumer electronics industry. According to the wearable technology database [3], there are currently 41 (medical), 77 (fitness) 117 (lifestyle) and 26 (entertainment) devices already available on the market.

Typical envisioned applications range from the medical field (e.g., vital sign monitoring, automated drug delivery, *etc*.), to entertainment, lifestyle, gaming and ambient intelligence. However, with regards to applications such as disaster, rescue and critical missions, workers safety in harsh environments (e.g., oil and gas fields, refineries, petro chemical and mining industries) as well as roadside and building workers, wearable WBAN technology can also play a vital role to not only save human lives but also to protect critical and valuable assets. In this paper, we will emphasize this new application paradigm for WBANs.

**Figure 1.**
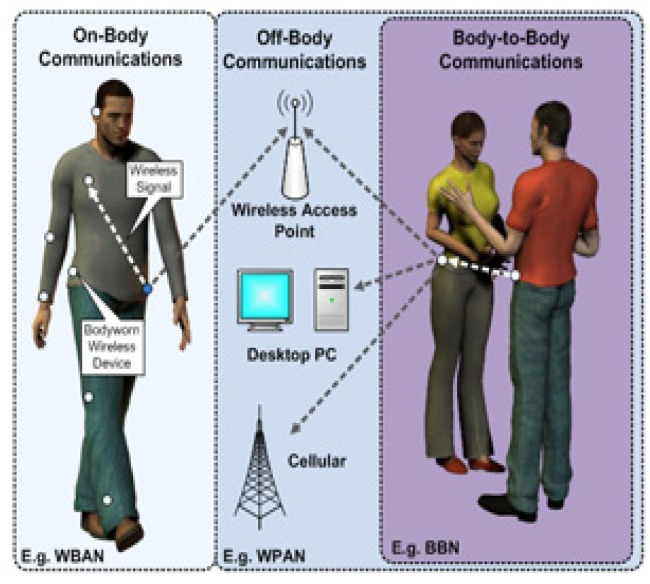
Overview of On-Body, Off-Body and Body-to-Body Communications.

WBANs are a variant of Wireless Sensor Networks (WSNs) which consist of a few tiny sensors implanted inside the body or located on the body to typically observe physiological signals emanating from different body organs, body motions as well as the surrounding environment. The network is designed in such a way that the coordinating device communicates with implanted and on-body sensors as well as with the access point which further transmits the collected information to a remote monitoring station. This is a typical scenario where only one body constitutes a WBAN, which is controlled by a more powerful coordinator (in comparison to other nodes in WBAN), and hence communicates with the distant off-body networks. We term this system a ‘*closed-system*’ because one WBAN cannot communicate directly with other WBANs, whereas, in the context of rescue and critical mission-based applications, ‘*closed-system*’ WBANs are not enough because body-to-body communications is a key requirement to enhance the communication and reliability of the system. Therefore, in this paper, we classify WBANs as Intra-WBAN (or On-Body), Inter-WBAN (or Body-to-Body) and Beyond-WBAN (or Off-Body) networks, as shown in Figure 1.

Low-power devices attached on the body are constrained by minimum energy requirements due to their limited battery life and should be small enough in size to be easily wearable. The management of such a heterogeneous network (both from the different devices as well as from the highly dynamic traffic points-of-view) adds another dimension to the design issues [4]. Moreover, for the given applications, harsh environment, Quality-of-Service (QoS) and reliability of successful communication further add to the stringent constraints of the overall system. Therefore, these new applications require a thorough and comprehensive application-oriented and related issues-based review of the existing state-of-the-art, including standards and protocol stacks. In addition, it is necessary to analyze what are the key limitations of the existing solutions and finally to understand the future potential opportunities and challenges in this context.

It is important to highlight the main contributions and key differences in comparison to existing WBAN surveys. Among them, some well cited survey papers such as [5,6], were published a few years ago, and presented general overviews on WBAN requirements and design challenges for medical related applications, from the physical (PHY), medium access control (MAC) and networking layer points of view. More recently, Movassaghi *et al.* [7] surveyed medical and non-medical applications considering the latest development in the different layers, with special emphasis on routing and security. An interesting comparative analysis of existing radio technologies was explored in [8] to better understand the corresponding requirements of WBAN applications. Furthermore, it also presented specific issues concerning channel modeling, energy consumption, and coexistence. However, the scope of the applications covered by all these surveys is limited to closed-system with almost no Body-to-Body (or Inter-WBANs) interactions.

These general-purpose or medical WBANs solutions are very different from rescue and critical based applications, discussed in this paper, in terms of both design requirements and constraints. First, mobile workforces are generally deployed in harsh or disaster environments and require effective communication, coordination, and monitoring capabilities. Second, during practical cases of deployment existing infrastructure might be absent, damaged or overloaded and mobile workforces might face various uncertainties and hazards, and finally, sensitive and/or real-time information should still be transmitted from the deployed mobile workforces teams to external command centers to ensure timely and effective decision making. In this context, the resulting Wearable WBAN systems for safety and life critical applications should be autonomous, self-organizing, large scale, robust to external interferences, energy efficient, secure, and support various data types and rates. Moreover, these systems should enable cooperative (or collaborative), reliable and real-time communications while providing various quality of services (QoS) and security levels.

To the best of our knowledge, this survey paper is the first to address rescue and critical based applications in which wearable technologies based systems (*i.e.*, WBANs) are exploited. In typical WBAN research studies, health-care and monitoring of patients is considered as the main application. In contrast, this work explores the use of wearable WBAN technologies for rescue, mission-critical and worker safety applications. The detailed requirements and architectures of these applications are provided first, and the limitations of existing research and development in this context are highlighted. Moreover, the relevant key features of the newly published IEEE 802.15.6 WBAN standard [9] are investigated, along with a recent overview on WBAN research in order to help researchers and developers rapidly understand the key requirements and design challenges of such applications. Finally, a case study (worker safety and protection) is discussed as one of the potential applications from the open research issues and challenges points-of-view.

The rest of this paper is organized as follows: after this Introduction, WBANs for life and safety critical applications are presented in Section 2, which includes key technologies, detailed specific applications and requirements, and finally the limitations of the existing solutions with regards to the given applications are discussed. The key enabling IEEE 802.15.6 standard and its important features such as physical layer details, medium access mechanisms, coexistence, and security, are explored in Section 3. A recent and detailed state-of-the-art on the WBAN communication stack, including PHY, MAC and networking layers, is provided in Section 4. In Section 5, existing research projects and their limitations are presented, and open research issues and challenges are discussed as an inspiration for the future works.

## 2. WBANs for Life and Safety Critical Applications

There is currently a growing need for ubiquitous communication and monitoring systems enabling life and safety critical applications. Indeed, these application areas are currently attracting a lot of attention in the research and industrial community. In this context, it is envisioned that wearable human assistive technologies will play an important role in managing, monitoring, and ensuring the safety of humans during mission critical operations.

### 2.1. WBANs—The Key Enabling Wearable Technology

With the recent advances in electronics and the development of short range and ultra-low power radio technologies, e.g., Zigbee, Bluetooth Low Energy (BLE), Ultra Wideband (UWB), and so on, Wireless Body Area Networks (WBANs) have recently emerged as a key enabling technology for many applications.

A WBAN consists of multiple miniaturized, smart and self-powered sensor devices that can be attached, or even implanted into, humans to monitor their physiological parameters (e.g., temperature, blood pressure, heart pulse rate, *etc*.), body motion (e.g., posture, orientation, location, *etc*.) as well as their surrounding environment (e.g., toxic gases, humidity, heat, *etc*.). All the monitored parameters are then transmitted wirelessly, using short-range on-body or Intra-WBAN communications, from the on/in-body sensors to the WBAN's coordinator for further data processing and analysis.

The WBAN coordinator is generally considered as a more resource-rich device that can wirelessly interconnect the WBAN sensors to external network infrastructures, using off-body or beyond-WBAN communications, such as static Wireless Sensor Networks (WSNs), WiFi Access Points or Broadband Cellular Networks (e.g., GSM, GPRS, 3G, LTE, *etc*.). In case of unavailable or out-of-range network infrastructures, the WBANs coordinators and/or WBANs sensors can exploit cooperative and multi-hop body-to-body or inter-WBANs communications to extend the end-to-end network connectivity, thus forming a self-organizing and dynamic *Wireless Body-to-Body Network* (WBBN).

As shown in Figure 1, the resulting WBANs communication architecture is based on three main tiers: (i) *Tier-1*: which correspond to the Intra-WBANs communications between the on-body sensors and the WBAN coordinator; (ii) *Tier-2*: which correspond to the beyond-WBANs communications between the WBANs coordinators and the outside world using, for example, available cellular networks or network infrastructures (e.g., WiFi, WSNs, *etc*.); and (iii) *Tier-3*: which correspond to the inter-WBANs communications between neighbor WBANs.

### 2.2. Applications of WBANs

WBANs span a wide range of target applications such as the rescue and emergency management in disaster areas [10–14], the monitoring of mobile workforces operating in harsh or unhealthy environments [15–19], the monitoring and tracking for personal healthcare [20,21], and many other applications. This section focuses on safety and life critical applications and describes their main characteristics and requirements.

#### 2.2.1. WBANs for Rescue and Emergency Management

The growing numbers of disasters and accidents, in terms of frequency and intensity, has a significant impact on humans' living conditions, asset safety, as well as the economy. These disasters might be caused by a combination of either natural, man-made or unexpected factors and their negative impact on human beings is amplified due to the increased population densities in cities, public areas, or buildings. For instance, fire is responsible for the largest number of deaths in the US in comparison with all other natural disasters combined. Indeed, more than 1 million fires were reported in the US in 2012 [22], and which caused 2,855 civilian deaths, 16,500 civilian injuries and $12.4 billion in property damage. In this context, it is envisioned that WBAN technology will enable humans to respond to these unexpected events in a timely and efficient manner in order to reduce injuries, deaths, asset and economic losses [10–14].

**Figure 2.**
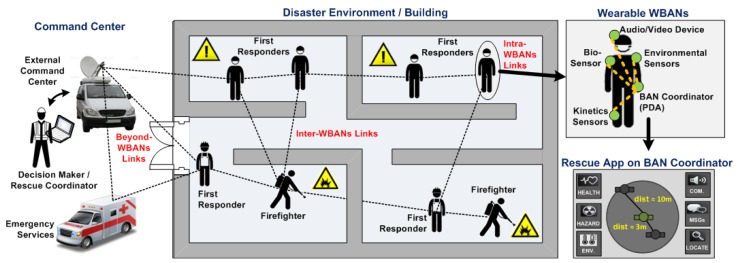
WBANs for Rescue and Emergency Management Application Use-Case.

As shown in Figure 2, during a disaster response scenario, field personnel (*i.e.*, first responders [14], emergency personnel [10], firefighters [13], *etc*.) can face various uncertainties and hazards on their operational theaters, such as no *a-priori* knowledge of the people or assets to be rescued, limited or zero visibility, the unavailability of communication infrastructure, very high ambient temperatures, the emanation of toxic gases, and many other risks.

By fitting field personnel with wearable WBANs technology, decision makers and corresponding authorities will be able to gather accurate and real-time information from these field personnel in order to better anticipate and manage rescue and life-critical operations. The gathered information can be related to the field personnel's health status (e.g., heart pulse rate, stress level, blood pressure, *etc*.), their location and movement inside harsh or damaged environments (e.g., absolute and/or relative location, body posture and orientation, *etc*.), the environmental conditions (e.g., heat, toxic gases, lightening, fire, smoke, *etc*.) and the status of their ongoing tasks (e.g., search, recovery, evacuation, assistance, *etc*.). The efficient coordination, remote monitoring, and communication with/between these teams and their command centers are thus one major objective yet to be achieved. Moreover, it is also envisioned that WBANs will also enable emergency personnel to better manage the safety and health of rescued patients, for example, by enabling in-field patient triage and health monitoring prior to their arrival at the hospital [10].

#### 2.2.2. WBANs for Mobile Workforce Safety and Health Management

With the expansion and emergence of large mega construction projects, the safety and health of workers is also becoming a serious concern worldwide. For instance, according to the World Health Organization (WHO), the number of deaths due to work-related accidents or diseases, remains unacceptably high at around 2.3 million per year [23].

In this context, it is expected that WBAN technology will enhance the safety and health of workers, for example, by enabling the remote monitoring of workers in unhealthy environments [18] as well as their activities [15] and physiological load [19], the tracking of mobile workers' positions to notify them about possible hazards (e.g., approaching trains [16], *etc*.), the protection of construction workers against carbon monoxide poisoning [17], and many others applications.

To that end, each worker will be fitted with a set of wearable sensing devices to continuously monitor their: (i) *physiological parameters* (e.g., energy expenditure, heart pulse rate, stress level, *etc*.); (ii) *location* (e.g., GPS position, relative proximity to hazards, *etc*.); (iii) *body movement* (e.g., body posture and orientation, activity, fall detection, *etc*.); and (iv) *surrounding environment* (e.g., toxic gases, CO_2_ or CO, heat, *etc*.). All the collected information are then processed locally and/or remotely to better anticipate and protect the deployed mobile workforces from accidents and hazards, thus reducing injuries and fatalities and improving working conditions.

**Figure 3.**
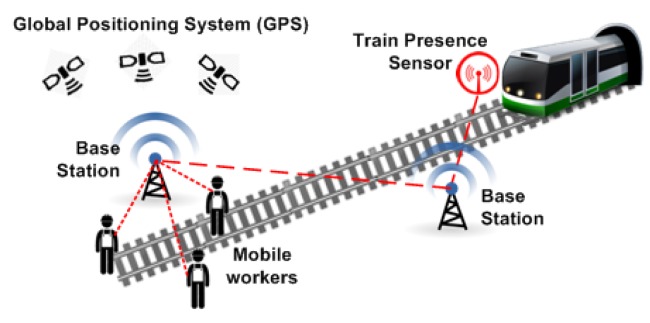
WBANs for Mobile Workforce Safety and Health Management Application Use-Case 1.

**Figure 4.**
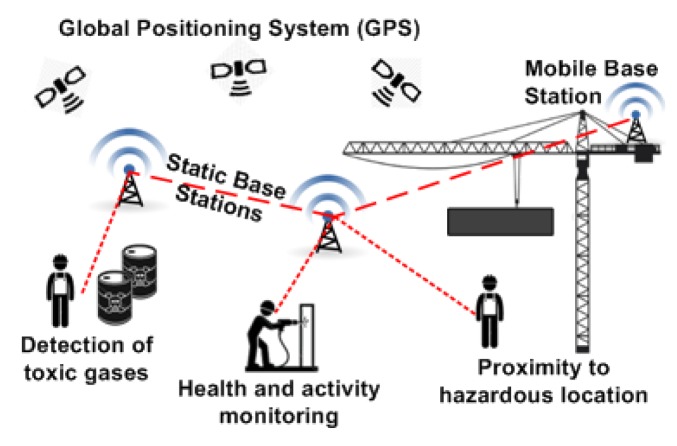
WBANs for Mobile Workforce Safety and Health Management Application Use-Case 2.

Two typical application scenarios for mobile workforce safety and health management are shown in Figures 3 and 4, respectively. The first scenario illustrates a safety-critical track warning system [22] where base stations are deployed along a railway to detect approaching trains, and to notify trackside railway workers through wearable and real-time communication devices, thus improving their safety. The second application scenario illustrates a safety and health management system for construction workers, where each worker is fitted with wearable sensing and communication devices to protect them from the presence of potentials hazards and to better anticipate health-related issues.

### 2.3. WBAN Requirements

In order to address the increasing demand for Wearable Human Assistive solutions, the IEEE 802.15.6 (TG6) task group has recently approved and released a WBAN IEEE standard [9] for short-range wireless communications on, or inside, human bodies, and whose functional requirements are summarized in Table 1. Given the wide range of target WBAN applications the IEEE 802.15.6 norm was designed with several key requirements in mind, such as the achieved data rates, power consumption, network size and security. Indeed, the main requirements of the IEEE 802.15.6 are summarized as follows [7,9,24]:

- The communication range of the WBANs nodes should be around a few meters (typically 3 m) under low data rates, and it should support the one-hop or two-hop star topology;
- WBAN nodes should be able to be dynamically inserted and/or removed from the WBAN system in less than 3 s;
- WBANs should support up to 64 nodes per WBAN, and up to four WBANs located on the same body (*i.e.*, a total of 256 nodes per body);
- Due to the high heterogeneity of WBAN applications and on/in-body sensors, the typical data rates of the WBAN communication links should be between 10 Kb/s and 10 Mb/s depending on the considered application, as shown in Table 2;
- The achieved packet error rate (PER) should be less than 10%, assuming a data payload of 256 bytes, with a link success probability of 95% over all channel and movement conditions;
- The achieved communication latency should be less than 125 milliseconds for medical applications and less than 250 milliseconds for non-medical applications, whereas the jitter should be less than 50 milliseconds;
- The WBAN communication links should be reliable and support different quality of services (QoS) by providing, for example, fast alarm messages delivery (e.g., in less than 1 s) during emergency situations. Moreover, periodic and burst traffic patterns should be supported;
- The WBANs should support ultra-low power consumption, with devices able to operate from several hours up to several years depending on the target applications. For example, WBAN nodes should be able to operate for more than 1 year using a 500 mAh battery and 1% low duty cycle (LDC) protocols;
- Up to 10 WBANs should be able to coexist in a volume of 6 m³, and should be robust against the presence of other heterogeneous communication standards and interferences, such as WiFi or Bluetooth;
- WBANs should support low-overhead and lightweight security features, such as the authentication of the WBAN devices, data integrity, and data encryption;
- Finally, WBANs devices should be ergonomic, in terms of sizing, functionalities, and lifetime, in order to enable the emergence of future body centric applications.

### 2.4. Limitations of Existing WBANs Solutions

Despite the specific constraints and requirements of Wearable WBANs (*cf*. Table 1), existing communication technologies, and standards are currently still used for the design of short-term and ready-to-use WBAN solutions [25]. This is mainly due to the current unavailability of commercial and off-the-shelf IEEE 802.15.6-compliant devices.

These standards include *Personal Area Network* (PAN) technologies, such as Bluetooth and Bluetooth Low Energy (BLE), *Wireless Sensors Network* (WSN) technologies, such as Zigbee (IEEE 802.15.4) and Ultra Wide Band (IEEE 802.15.4 a), and *Wireless Local Area Network* (WLAN) technologies, such as Wifi (IEEE 802.11 a/b/g/n).

**Figure 5.**
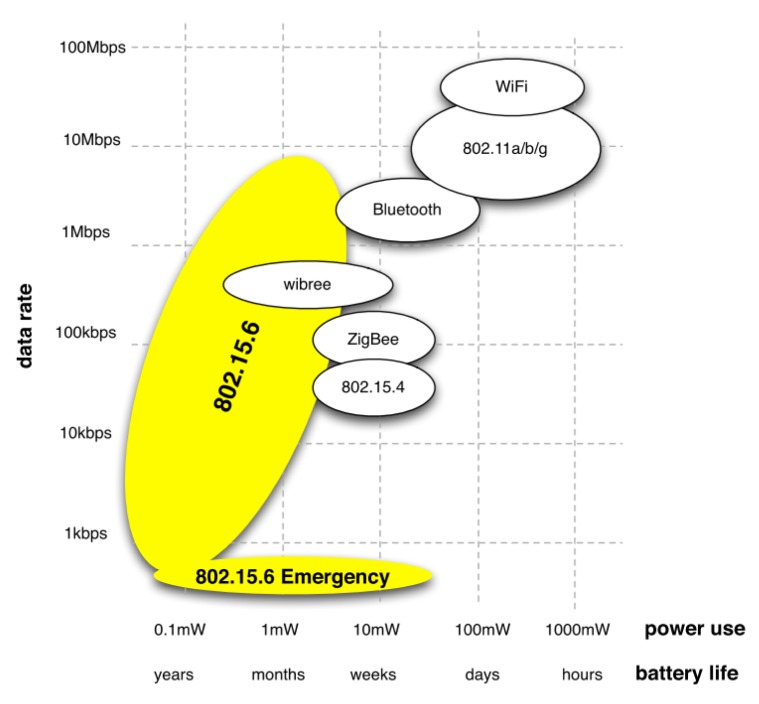
Power and Data Rate Requirements for the IEEE 802.15.6 WBAN Standard [9].

However, since these communication standards were designed with other applications in mind, they do not always meet the specific requirements and constraints of WBAN applications, and present major limitations in terms of peak-power consumption, achieved data rates, communication range, generated RF interferences, and efficient on-body communications, as shown in Table 3. More specifically, the following main differences can be highlighted between WBANs and existing PANs, WSNs and WLANs related applications:

- The power consumption of existing standards is too high in comparison to the specific requirements of WBAN applications. As shown in Figure 5, power levels are in the range of 800 mW for Wifi, 100 mW for Bluetooth, 50 mW for Zigbee, whereas WBANs require lower power levels of around 0.1–1 mW to increase the battery and network lifetime;
- Existing communication standards were not optimized for body-centric communications, especially regarding WBANs' inherent intra- and inter-mobility patterns and the RF propagation around, or inside, the body;
- The communication stacks of existing standards do not meet the specific requirements of WBAN applications in terms of reliability, low power consumption, high data rates and robustness against external interferences;
- Finally, existing communication modules are still too large to fit in miniature housings, which is a key requirement for most WBAN applications.

Finally, there are number of challenges and issues that need to be addressed before WBANs can be effectively utilized for the new applications. Later in Section 5, those issues, challenges, and opportunities are discussed in detail. In the next section, some of the key attributes of the WBAN standard (*i.e.*, IEEE 802.15.6) which are necessary for rescue and critical applications are explored and presented.

## 3. IEEE 802.15.6: The Key-Enabling Standard for WBANs

The main challenge in designing standards for WBANs is the balance of QoS demands with the low power constraints of limited battery powered nodes [30]. More specifically, for human assistive rescue and critical missions, and workers safety in hazard-prone area-based applications, WBAN system requirements may not be the same as those of classical healthcare systems. For these applications, since the sensor devices attached on the body are required to be small, therefore, they have to be low-power and energy efficient. In addition, most importantly, reliability and QoS with high data rates are the crucial requirements for the given applications.

Various low power standards have been used in WBAN research as well as for commercial applications, where most of them partly satisfying the requirements for life signs monitoring as already discussed in Section 2.4. Some low power standards designed to support low power sensing that have been adapted for healthcare applications, e.g., ZigBee, while others such as IEEE 802.15.6 have been designed specifically for WBANs, not only for mobile healthcare monitoring, but also for many more applications. Due to the greater flexibility, high data rates, better security and reliability and higher QoS, it fits well to the requirements of the WBANs. In this section, we will explore in detail the IEEE 802.15.6 standard, including physical (PHY), medium access control (MAC), network topologies and security features.

### 3.1. A Brief History of IEEE 802.15.6 Standard

In November 2007, the IEEE 802.15 Task Group 6 (also known as IEEE 802.15.6) was formed in order to standardize Wireless Body Area Networks (WBANs), which were not covered by any existing communication standard by then and later in 2012, the final version of the standard has been released as presented in [9]. The standard defined Physical (PHY) and Medium Access Control (MAC) layers optimized for short-range transmissions in, on, or around the human body. The purpose is to support a low complexity, low cost, ultra-low power, and high reliable wireless communication for use in close proximity to, or inside, a human body (but not limited to humans) to serve a variety of applications both in the medical/healthcare and in the non-medical fields. In the following sections, we will explore more details of the standard and its important features.

### 3.2. IEEE 802.15.6 Physical Layer

**Figure 6.**
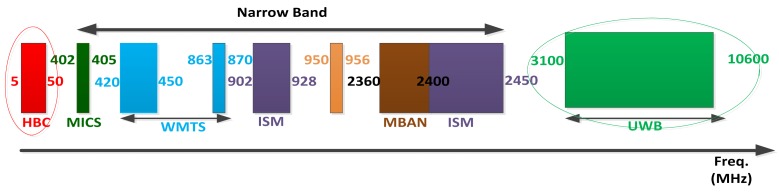
Radio Frequency Spectrum for WBAN Communications [9].

The graphical summary of the spectrum allocation of all available frequencies for WBANs applications is shown in Figure 6. The IEEE 802.15.6 standard has proposed three different alternatives for the PHY layer based on the specific applications and their requirements, including Human Body Communications (HBC), Narrowband (NB) PHY and Ultra wideband (UWB) PHY, which are as explained below:

- ***Human Body Communications (HBC) PHY***: IEEE 802.15.6 introduced a new communication medium (HBC) for the frequency range of 5–50 MHz for implanted devices and their interaction with coordinating device. Its primary technology is electric field coupling which includes capacitive coupling and galvanic coupling. The HBC channel of the capacitive coupling type is developed based on the near electric field around the human body, which is induced by a transmitter terminal, and a receiver terminal is used to detect the weak coupling changes of the near electric field along the body channel. Research has shown that the data rate of capacitive coupling is up to 2 Mb/s. Two bands of operation cantered at 16 MHz and 27 MHz with a bandwidth of 4 MHz are proposed in the standard.
- ***Narrow Band (NB) PHY***: In NB communication multiple nodes are on the body to communicate with each other and normally it is not used for implanted devices due to relatively high power consumption. A compliant device shall be able to support transmission and reception in one of the following frequency band:
  ○ 402–405 MHz: Medical Implant Communication System (MICS) band, it is widely accepted although the possible bandwidth is limited;
  ○ 420–450 MHz: Wireless Medical Telemetry System (WMTS) band (available in Japan);
  ○ 863–870 MHz: WMTS band (available in Europe);
  ○ 902–928 MHz: ISM band, it is available for use without a license in North America, Australia and New Zealand;
  ○ 950–956 MHz: Available in Japan;
  ○ 2360–2400 MHz: This frequency band is proposed by the standardization group to be adopted in BAN applications;
  ○ 2400–2483.5 MHz: ISM band, it is available worldwide, but there could be issues of coexistence with other IEEE standards that use the same band;
- ***Ultra Wide Band (UWB) PHY***: In UWB the frequency range varies between 3.1 GHz and 10.6 GHz. It provides high performance, robustness, low complexity and ultra-low power operations for WBANs. It is available worldwide while some spectral masks are defined differently in different countries or regions.

A typical WBAN is usually composed of a few body sensor nodes, even though up to 256 nodes are supported by the IEEE 802.15.6 standard. A relatively wide range of data rates can be employed; varying from 1 kbps to 1 Mbps, which is one of the most important features while considering the wide range of target WBAN applications.

**Figure 7.**
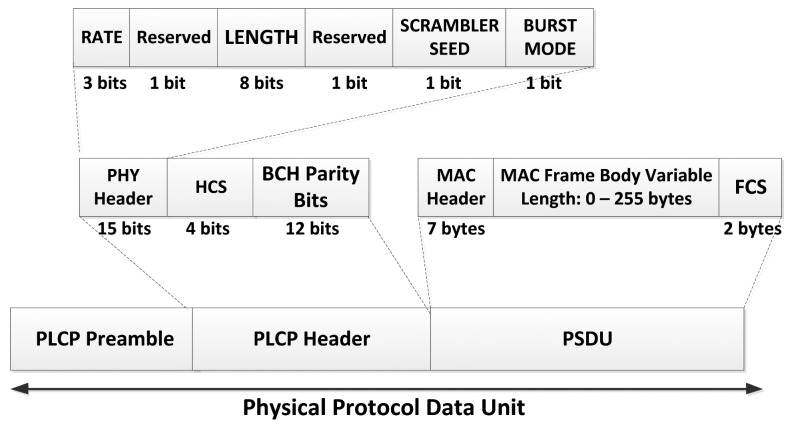
Physical Frame Format [9].

The Physical-Layer Protocol Data Unit (PPDU) represents the information that is sent through the propagation medium to the receiver device. It is composed of the physical layer convergence protocol (PLCP) preamble, physical layer convergence protocol (PLCP) header, and physical layer service data unit (PSDU), as illustrated in Figure 7, and is briefly explained below:

- ***PLCP Preamble***: The purpose of the preamble is to aid the receiver in packet detection, timing synchronization and carrier-offset recovery. Two unique preambles are defined in order to mitigate false alarms due to other networks operating on adjacent channels. The preamble is transmitted at the symbol rate for the desired band of operation and will be encoded using the same modulation parameters as defined for different physical types. More details on the PLCP Preamble can be found in section 8.2 of the standard [9].
- ***PLCP Header***: It is added to convey information about the PHY and MAC parameters that are needed at the receiver side in order to decode the PSDU. Details on the different parts composing the PLCP Header and how to properly set the bits of each field can be found in section 8.3 of the standard [9].
- ***PSDU***: It is formed by concatenating the MAC header with the MAC frame body and Frame Check Sequence (FCS). The PSDU is then scrambled and optionally encoded by a BCH code. The PSDU shall be transmitted using any of the available data rates in the operating frequency band. More details on PSDU construction can be found in [9].

### 3.3. IEEE 802.15.6 MAC Layer

There are three different types of nodes in typical WBANs, including one or more coordinators (which is normally more powerful in terms of energy and processing capabilities), then a sensor node (power-limited) and finally a relaying node (where a coordinator can be a relaying node as well). MAC layer is the core of the standard as it provides greater flexibility to the users to enable hybrid *Time-Division Multiple Access* (TDMA), *Carrier Sense Multiple Access/Collision Avoidance* (CSMA/CA) and the combination of both) medium access mechanism to meet the required applications requirements. Below we will explore detailed features and access mechanisms of the MAC protocol.

#### 3.3.1. MAC Frame Format

**Figure 8.**
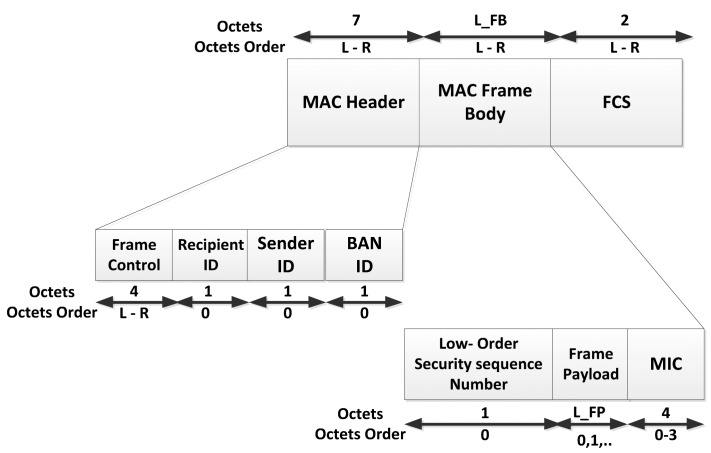
IEEE 802.15.6 MAC Frame Format [9].

The MAC Protocol Data Unit (MPDU) is an ordered sequence of fields delivered to or from the PHY Service Access Point (PHY SAP). The MAC frame consists of a fixed-length MAC header (seven octets), a variable-length MAC frame body and a fixed-length Frame Check Sequence (FCS) field (two octets), as shown in Figure 8.

The MAC frame body has an octet length *L_FB* such that *0 ⩽ L_FB ⩽ pMaxFrameBodyLength*, and is present only if it has a nonzero length, where ‘*pMaxFrameBodyLength*’ is the maximum frame body length at the physical layer. The Low-Order Security Sequence Number and Message Integrity Code (MIC) fields are not present in unsecured frames. Management, Control, and Data type are the three MAC frames that are described in detail in the standard. Each of them implies a different composition of the MAC Frame Body, in particular for the Frame Payload Field.

#### 3.3.2. MAC Access Mechanisms and Techniques

**Figure 9.**
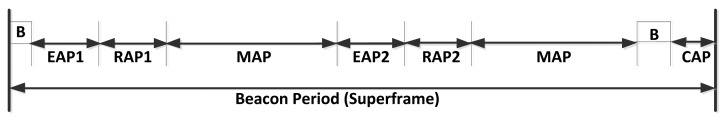
Layout of Access Phase in a Beacon Period (Superframe) for Beacon Mode [9].

**Figure 10.**
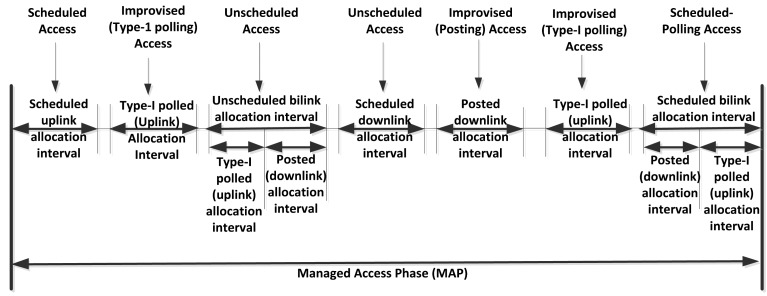
Allocation Interval and Access Methods Available in Managed Access Phase [9].

**Figure 11.**
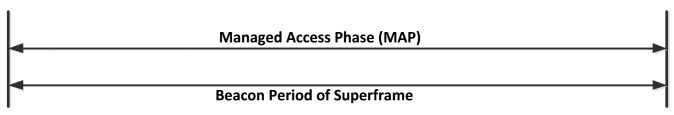
Layout of Access Phase in a Non-Beacon Superframe Boundary Mode [9].

**Figure 12.**
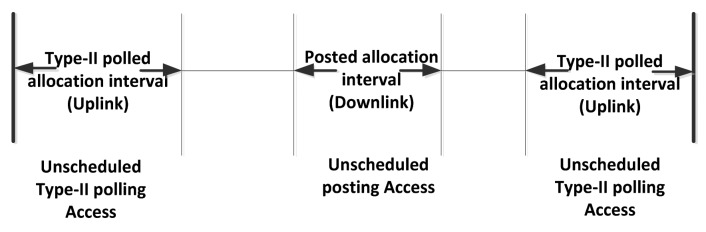
Non-Beacon With Out Superframe Mode [9].

The standard provides greater flexibility on the users to adapt MAC according to their requirements. With regards to medium access mechanisms, WBANs Coordinator can decide to operate in one of the following access modes:

- ***Beacon mode with superframe boundaries***: A beacon is transmitted at the beginning of every beacon period, as shown in Figure 9. In this way a common time base is established, to enable time referenced allocations. There are two Exclusive Access Phases (EAP; EAP is added in this standard to support the high priority and emergency traffic.) and two Random Access Phases (RAP, used for regular non-emergency traffic.), which can be configured together based on the application requirements as EAP1 and EAP2 respectively. For the medium access only contended allocation is possible which means either CSMA/CA or Slotted Aloha can be used during EAP, RAP and CAP periods. Whereas, during the managed access phase (MAP) there are scheduled, unscheduled and improvised access options as shown in Figure 10.
• ***Non-beacon mode with superframe boundaries***: In this mode beacons are not transmitted but superframe and allocation slots boundaries are established which provides time referencing to the medium access. Figure 11 shows the layout of the non-beacon mode with Superframe boundary, where it can be seen that only managed access is possible in this mode
- ***Non-beacon mode without superframe boundaries***: In this mode beacons are not transmitted, superframe and allocation slots boundaries are not established because there is no time reference involved in accessing the medium. Figure 12 shows the non-beacon mode without superframe medium access. It is based on unscheduled access with type-II polled uplink and post allocation for downlink by using CSMA/CA mechanism.

### 3.4. Network Topology

**Figure 13.**
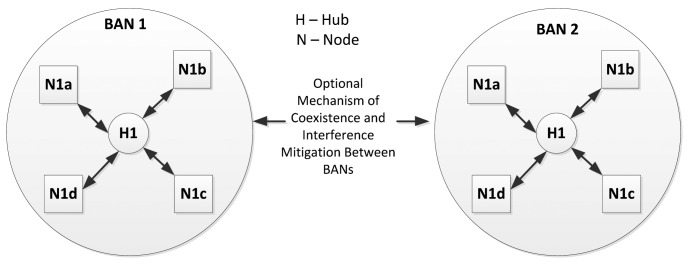
Network Topology (One-Hop Star) [9].

**Figure 14.**
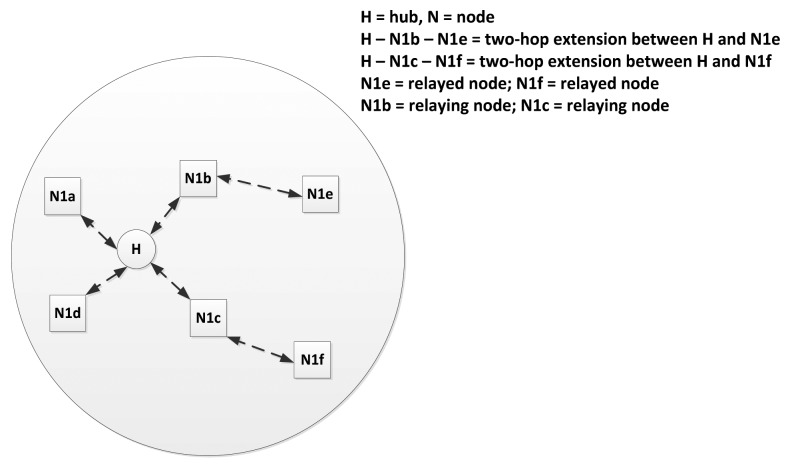
Network Topology (Two-Hop Extended Star) [9].

Typically, a number of sensor nodes and one coordinator are organized to constitute a WBAN. In a single WBAN, there is only one coordinator which is either directly connected to all the nodes through one-hop star topology (as shown in Figure 13) or through two-hop extended star topology (which is often called as Restricted Tree Topology as shown in Figure 14) to exchange the frames via a relay node. It should be noted that the two-hop extended star topology is restricted in Medical Implant Communication Service (MICS) band. The maximum numbers of nodes in a body area network are limited to ‘*mMaxBANSize*’ which varies for different applications.

### 3.5. Coexistence and Interoperability

In the IEEE 802.15.6 proposal, a WBAN is defined as a pico-area network, with a coordinator and a set of associated sensors in a TDMA mode. To ensure a real-time efficient transmission, the WBAN coordinator allocates, in the Contention Free Period (CFP), one dedicated time-slot for each sensor in the WBAN. In this case, as long as no other WBANs are present in the close vicinity, Inter-WBAN interference is avoided, reducing thus the packet error rate (PER) of Intra-WBAN communications. Nevertheless, when multiple WBANs are co-located, since none of them has the ability to stop the transmission of the others, the WBANs obviously interfere with each other (*i.e.*, Inter-WBAN interference).

Intra-WBAN and Inter-WBAN coexistence can be typically be classified as collaborative and non-collaborative. In collaborative coexistence, there is coordinator-to-nodes (for Intra-WBANs), and coordinator-to-coordinator (for Inter-WBANs), sharing of important information to avoid interference. Typical information includes the channel number, the operating frequency, the transmission power, *etc*. In non-collaborative coexistence, WBANs do not share any information for interference mitigation. Since the coordinator controls the Intra-WBAN communications, the different WBAN nodes have a low probability of operating at the same time over the same channel. However, chances of interference between WBANs are much higher in the non-collaborative case.

With reference to the MAC header of the general frame format of the standard, there is a field called ‘frame control’ consisting of four octets (as shown in Figure 8), which contains the key information about interference and interoperability as well as the various security levels. More details on the ‘frame control’ can be found in section 5.2.1.1 of the standard [9].

**Figure 15.**
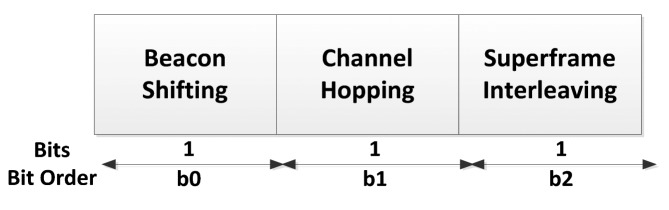
Coexistence Format of IEEE 802.15.6 [9].

The standard proposed three different non-collaborative techniques, according to the frequency band used and the mobility of the network that a coordinator could employ for coexistence and/or interference mitigation between Intra-WBANs and Inter-WBANs. These include *beacon shifting, channel hopping* and *interleaving* which are explained (in brief) below. The coexistence format (which is part of the frame control field of the MAC header) is illustrated in Figure 15.

The Beacon Shifting field is set to one if beacon shifting is currently enabled, or is set to zero otherwise. Similarly, the Channel Hopping field is set to one if channel hopping is enabled, or is set to zero otherwise. The super frame Interleaving field is set to one if the sender (a coordinator) supports active super frame interleaving and command frames, or is set to zero otherwise.

#### 3.5.1. Beacon Shifting

A coordinator may transmit its beacons at different time offsets relative to the start of the beacon periods by including a Beacon Shifting Sequence field in its beacons (specific sequence details can be found in section 5.3.1.10 of the standard [9]). A coordinator should choose a beacon shifting sequence that is not being used by its neighbor coordinators to mitigate potential repeated beacon collisions and scheduled allocation conflicts between overlapping or adjacent WBANs operating in the same channel.

#### 3.5.2. Channel Hopping

A coordinator may change its operating channel periodically by choosing a particular channel hopping sequence, which is not being used by any neighbor coordinators. Moreover, the coordinator should hop to another channel after dwelling on the current channel for a fixed number of beacon periods and not in the middle of a super frame.

A prospective WBAN coordinator should select a channel to operate prior to starting a new network, which could be done by performing a channel scan (*i.e.*, active, passive or energy detection based) over a given list of channels in a specific frequency band. At the end of the scan, the coordinator may select an unoccupied channel for network operation. If no free channels have been detected, the coordinator selects an occupied channel and uses the information gathered during the scan to operate in Time Sharing, Offset Synchronization or Interference Mitigation mode. Further details on channel hopping can be found in section 6.13.2 of the standard.

#### 3.5.3. Interleaving

A WBAN may share the same operating channel with one or more other WBANs with or without interleaving their active super frames. A coordinator that supports active super frame interleaving and operates in non-beacon mode with super frames sends a B2 frame in every active super frame to avoid channel interference. More details on Interleaving are provided in Section 6.13.3 of the standard.

### 3.6. Security

The IEEE 802.15.6 standard provides both secure and unsecured communications. A node and a coordinator follow several different states at the MAC level for secured communication. In secured communication there are three states that include Orphan, Associated and Secure states whereas, only Orphan state in unsecured communication. More details on these states can be seen in section 4.5 of the standard [9]. All nodes and coordinators are offered three security levels:

- ***Level 0—unsecured communication***: Messages are transmitted in unsecured frames, which provide no measures for message authenticity and integrity validation, confidentiality and privacy protection, and replay defence.
- ***Level 1—authentication but not encryption***: In this level, messages are transmitted in secured authenticated but not encrypted frames, which provide measures for message authenticity and integrity validation and replay defence but not confidentiality and privacy protection.
- ***Level 2—authentication and encryption***: The messages are transmitted in secured authenticated and encrypted frames, which provide measures for message authenticity and integrity validation, confidentiality and privacy protection, and replay defence.

During the association state, a node and a coordinator should jointly select a suitable security level for their subsequent secured frame exchanges. In addition, based on their respective security requirements and certain information specific to each other, they should check whether authentication is required. For unicast secured communication, the node, and the coordinator shall activate a pre-shared Master Key (MK) or establish a new MK via an unauthenticated or authenticated association, and shall create Pair-wise Temporal Key (PTK) via a PTK creation procedure. For multicast secured communication, the coordinator shall distribute a Group Temporal Key (GTK) to the corresponding multicast group.

**Figure 16.**
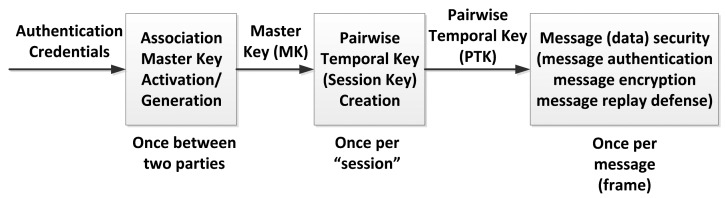
Security Hierarchy [9].

The node and the coordinator shall follow the security hierarchy shown in Figure 16 to perform security key generations and provide message security services. A session indicated in this figure, refers to a time span in which a temporal key remains valid.

After elaborating the necessary and ley features of the WBANs standard, in the next section a comprehensive and latest study on communication stack ranging from mobility and radio channel modeling to MAC and Networking algorithms and protocols is presented.

## 4. The WBANs Communication Stack

The communication stack of WBANs is mainly governed by the application layer, which specifies a number of important parameters such as the packet transmission rate, the network topology, the traffic patterns, the required quality of service, and so on. Moreover, the selection of appropriate routing strategies, feasible medium access mechanisms, available physical layers, channel and mobility modeling, are important stages during the design of WBAN-based applications. This section explores the most recent research works on the mobility and channel modeling for WBANs, as well as the design of efficient MAC and networking layers for WBANs. The reader is referred to Section 3 for further details about available IEEE 802.15.6 physical layers and their related parameters.

### 4.1. Mobility and Radio Channel Modeling for WBANs

In order to realize optimized body centered communication between wearable sensors, understanding the behavior of wireless channels is very important. The channel model generally characterizes the path loss of WBAN devices that takes into account shadowing due to the human body mobility or obstacles near the human body and postures of human body. According to the latest development in channel modeling for WBANs, IEEE 802.15.6 proposed number of channel models in [31] and explains the following main differences from typical wireless channel [32]:

- ***Type of antennas***: Electric type antennas such as dipoles and magnetic type antennas such as loops interact with the human body differently. In general, the magnetic type antennas are known to generate weaker electric fields in human tissue so that the specific absorption rate (SAR) is smaller. As well, the radiation pattern and the polarization are different for these two types.
- ***Setup of body area devices/antennas***: Position of the body influences the orientation of antennas and shadowing due to the different parts of the body. Rotation of the devices/antennas causes the change of the directions of radiation pattern and polarization. Small spacing between the body and the antenna causes the impedance mismatch and the distortion of the radiation pattern.
- ***Posture and movement of body***: Depending on the posture and the positions of the devices/antennas, part of the body may shadow the line-of-sight (LOS) path. In particular, the shadowing by the trunk causes very large attenuation [33]. Movement of the body changes the orientation of the antennas, distances between antennas, and the shadowing conditions.
- ***Variation of human body***: The channel response varies with different human samples. Many factors such as the difference in the shape and the dielectric constant may influence the channel response.

The next section summarizes the most important characteristics and key features of the IEEE 802.15.6 proposed channel models.

#### 4.1.1. Proposed IEEE 802.15.6 Channel Models

The WBAN radio channel is mainly impacted by two main phenomena, *i.e.*, small-scale fading and large-scale fading:

*Small-scale fading* refers to the rapid variations in the phase and amplitude of the signal during propagation over a small-area due to small changes in the location of the on-body device or body position/postures. It is mainly caused by small displacements of the antenna on the body and gross/minor changes in the body position/posture.
*Large-scale fading* refers to the fading due to motion over large areas; this is referring to the distance between antenna positions on the body and external node (home, office, hospital, *etc*.). It is mainly caused by large displacement of the body and to major changes in the body position/posture.

The IEEE 802.15.6 standard proposes different channel models for three different physical types (*i.e.*, Human Body Communication (HBC), Narrow Band (NB) and Ultra Wide Band (UWB). It defines three different types of nodes with reference to channel models (*i.e.*, implant, body surface, and external nodes). In addition, it describes different scenarios (as shown in Table 4) for same frequency bands, which is further, sub-divided into Line-Of-Sight (LOS) and Non-Line-Of-Sight (NLOS) links.

In the context of safety and critical applications, scenarios S4 to S7 can be considered for the accurate mobility and radio modeling, whereas scenarios S1 to S3 are normally reserved for medical based applications. For example, in case of narrow band, two models are proposed to satisfy scenarios S4 and S5. The authors of [34] proposed a model based on measurements (that cover frequencies of 2.4–2.5 GHz.) conducted in hospital room and anechoic chamber (to remove the multipath effects). The path loss function is defined as follows:

(1) $$PL\left(d\right)[dB]=a.\log_{10}(d)+b+N$$

where *a* and *b* are the coefficients of the linear fitting, *d* is the distance between (transmitter and receiver in mm), *N* is the normally distributed variable with standard deviation *σ*.

Typical values of the parameters of the path loss model are provided in Table 5. The path loss model is derived using a regression line through least square fitting for each frequency band. A transmitter antenna is placed at the waist and a receiver antenna is placed on different parts of the body, including head, ear, shoulder, wrist, waist, leg, and ankle.

The IMEC Research Center provided real-time measurements at 2.45 GHz of frequency [35]. The measurement setup includes antennas of 50.5 × 28 × 8 mm size with a weight of 4.2 g. Three planes separated by approximately 15 cm along the vertical axis around torso of the body are considered. The transmitter is placed on the front and the receivers are placed at distances of 10–45 cm in steps of 5 cm measured around the perimeter of the body. The transmitter is worn at approximately shoulder height at one of two different positions. The receiver is placed directly below the transmitter at seven positions separated by 10 cm covering the range from the shoulder to the knees. Six different locations are considered inside an office room and at each of the locations, 49 points are arranged in a height of 7 × 7 square grid. Six cm is the separation distance between each array element (for 2.45 GHz) and 16 cm (for 915 MHz).

The resulting path loss is modeled as a combination of exponential decaying function of the distance and average indoor attenuation, which is expressed by the following formula:

(2) $$PL\text{(}d\text{)}[\text{dB}]=-10\log_{\text{10}}(P_{\text{0}}e^{-md}+P_{\text{1}})+\sigma_{p}n_{p}$$

where *P*_0_ is the average loss close to the antenna and will depend on the type of antenna, *m* represents the average decay rate in dB/cm for the surface wave travelling around the perimeter of the body, *P*_1_ is the average attenuation of components in an indoor environment radiated away from the body and reflected back towards the receiving antenna, *σ_p_* is the log-normal variance in dB around the average representing the variations measured at different body and room locations. This parameter depends on variations in the body curvature and antenna radiation properties at different body locations.

The small-scale fading is modeled by a Ricean distribution with K factor that decreases as the path loss increases. The delay spread is normally distributed. Table 6 summarizes the model and corresponding parameters.

It should be noted that the above channel models are not realistic since they do not take into consideration important effects such as body mobility and channel temporal as well as spatial characteristics. As the IEEE 802.15.6 channel models were finalized in the year 2009, since then there have been many updates and progress for more realistic channel modeling. The next section highlights the latest research works on WBAN mobility and channel modeling.

#### 4.1.2. State-of-the-Art on WBAN Channel Models

The body area channel is very different from other wireless channels in the sense that the antenna and human body is an integral part of the channel so that the directional channel modeling approaches (such as [36]) which are getting more popular in multi-antenna and ultra wideband systems cannot be applicable. However, latest work such as [37], which is explained in this section, exploited a small multi-antenna based WBAN system and has shown that channel capacity and the performance can significantly improve with multi-antenna systems in WBANs. The following section presents the latest work on WBAN channel modeling.

It is important in radio channel modeling for WBANs to take into account human mobility for a realistic representation of the propagation phenomena. Biomechanical modeling clearly plays a central role in WBAN mobility assessment. Modeling the human body motion during pedestrian motion -on its own- is a pretty old topic, already investigated in computer graphics and robotics. It is however still hardly covered in the context of on-body radio communications. In particular, the relationship between body mobility and physical radio abstractions is a rather recent field of research. In [38] for instance, a cylinder-based biomechanical model is proposed to generate realistic mobility patterns as a function of time under typical pedestrian mobility. The model provides the true dynamic positions of on-body nodes, along with the corresponding obstruction conditions per link for arbitrary deployment scenarios. This model has been coupled further with macroscopic pedestrian mobility models (e.g., applying a random Gauss-Markov model to the body centroid) to evaluate the performance of localization algorithms.

A dynamic channel model based on extensive measurement campaign with different human subjects is proposed in [39]. The channel model is based on a scenario-based approach, where a scenario represents a specific TX and RX antenna location on the body, an environment, and a human body mobility condition. The mean path loss was expressed as a function of the scenario and not as a unique model as a function of antenna separation. This model was proven to be closer to reality that a distance-dependent model. The model allows taking into account the variation for different bodies, highlighting the existence of two main phenomena in BAN channel. First, the slow fading variation which strongly depends on the antenna locations and movement, and represents the masking effect of the human body itself. Second, the fast fading which take into account the multi-path effect in indoor environment [39].

An experimental study on how the signal properties vary as the person moves is presented in [40]. In this work sideways arm movement and marching on the spot movements are considered. The results obtained were used to derive a model for the behavior of the Body Coupled Channel (BCC) under moving body conditions. It was found that different body movements result in significantly different BCC channel behaviors. The type and speed of movement was found to affect the channel properties. It was concluded that the sideways arm movement is accurately modeled by a Lognormal distribution whilst the marching-on-the-spot movement is modeled by a Generalized Extreme Value distribution.

Periodic characteristics of wireless body area networks wireless channels measured using custom hardware in the 900-MHz and 2.4-GHz bands are presented in [41]. Received signal strength indication (RSSI) values of both bands were simultaneously sampled at a rate of 1.3 kS/s. Results are exploited from the measurements campaign of WBANs, which shows that characteristics of WBANs channels (such as periodicity) can be used for reducing the power consumption of wireless communication. A new channel model is introduced to add periodicity on-top- of the existing IEEE 802.15.6 WBAN path loss equations (expressed as Equation (2) in previous sections). Parameters such as activity factor and location factor are introduced to estimate the modified model parameters. Finally, a strategy for exploiting the periodic characteristics of the WBAN channel is presented as an example, along with the power savings from using this strategy.

In later work on channel modeling, the authors in [37] suggested that the virtual Multiple Input Multiple Output (MIMO) can benefit in WBANs from the capacity gain and enhancement of the overall performance of on- and off-body links. They propose a 2 × 2 MIMO-based model that includes the coupling between wearable antennas and the body, the body dynamics, and a realistic environment. The coupling between wearable antennas and the body was obtained through simulations. Then, user dynamics have been used to include the influence of the body posture on the orientation of the antenna. Finally, a geometrically based statistical channel model adapted to WBANs has been used in a propagation environment (clusters of scatters) to perform bounces of the transmitted signal over randomly distributed scatters to obtain multi-path components and channel impulse responses.

For the case of on-body communications, the proposed approach considered that the received signals are resulted from the contribution of one on-body component and several multi-path components present in the environment and obtained from the channel model. A WBAN with patch antennas operating at 2.45 GHz and nine possible locations on a female body have been analyzed. An indoor scenario is considered, and the user is walking. Based on proper metrics, optimum 2 × 2 antenna placements are suggested. Concerning on-body communications, the MIMO capacity was estimated for selected configurations and shows that the best performance is obtained when the sink nodes (RXs) are on front and back of the body, and the data sensors (TXs) are on the head, with a capacity gain of 0.97, almost reaching the upper MIMO capacity bound. Concerning off-body communications, an average capacity gain of 0.84 was achieved.

After explaining the existing state-of-the art on WBANs channel models a comparative study is summarized as follows. Different parameters are considered to evaluate various models. This includes scenarios and environment, path loss, propagation effects, mobility, link conditions and its types. IEEE 802.15.6 proposed channel models provides the basic distance-based path loss static models without any time varying effects and correlations features. Concerning time-varying modeling aspects and characteristics, the authors of [38,39] exploited spatial and temporal variations under dynamic environment and hence realized more accurate model. Periodic make-and-break links between moving parts of the body, such as to-and-fro motions of the arms in walking conditions required periodic time varying channel model. In [41] the authors proposed a RSSI-based path loss model with an addition of periodic characteristics on top of IEEE proposed channel models. It also provides a generic method to rapidly develop different path loss expression depending upon the specific user mobility and activity.

To conclude on the channel modeling from the presented state-of-the-art, it can be noticed that most of the models are developed with medical health-care applications in mind, therefore, the indoor environment is an obvious choice, however with regards to rescue and critical operations the environment can be outdoors as well and therefore, would require more realistic and new measurement campaigns. Further, as shown in Table 7, the impact of Body-to-Body (or Inter-WBANs) mobility and signal propagation variations for given scenarios are required to investigate for the future development of the application-specific accurate channel modeling.

### 4.2. Medium Access Control Layer

The Medium Access Control (MAC) layer plays a critical role in optimizing most of the important design cost functions. For example, MAC controls the radio activity and hence optimizes the energy consumption of the node by timely turning on and off the radio transceiver which is the most energy consuming component of the sensor node [42]. It provides medium access mechanisms such as time-based or random access to meet the time constraints (*i.e.*, latency optimization), and it provides the reliability of the successful transmission of packets by controlling the medium contentions.

In the WBAN context, the coordinator controls most of the communication and therefore TDMA is considered as an obvious choice for medium access. However, we will explore several medium access mechanisms while keeping the viewpoint of specific applications of rescue and critical operations as described earlier.

#### 4.2.1. MAC Protocols Design Principles for WBAN

Typically, several sensors connected on the body to monitor physiological as well as environment data layer to meet different time constraints according to various payloads (*i.e.*, data rate). It also reflects un-even energy consumption from a WBAN network from the lifetime point of view. There is also a cross layer inter-dependency at the MAC level, as the nodes that have more data to transmit should have more access to the channel (such as more slots in a TDMA frame) to meet their time constraints and therefore will consume more energy. Similarly, specific network topology (*i.e.*, star, one-hop or two-hop star for the intra-body, multi-hop for the inter-body and mesh for off-body communication) also have impact on the MAC-layer design. In this regards, different medium access mechanism could be required such as TDMA for intra-body, hybrid access for inter-body and off-body communications. Before going into the details of the existing MAC protocols below section will highlight important design constraints for application-specific WBAN.

#### 4.2.2. Key Attributes for Application-specific WBAN MAC

With regards to the energy consumption and lifetime of the WBAN devices, according to IEEE 802.15.6 TG6 Technical Requirements [43], the device should operate while supporting a battery life of months or years without intervention, whereas others may require a battery life of tens of hours due to the nature of the applications and/or physical constraints on the size of the devices. For example, cardiac defibrillators and pacemakers have a lifetime of more than 5 years, whereas swallowable camera pills typically have lifetime of 12 h. Most non-medical applications have stand-by power requirements of 100–200 h and active power requirements of several hours.

Most of the WBAN-based MAC layer protocols and surveys have considered energy consumption as the most important design constraint [44–46]. For example, for classical health monitoring applications such as ECG, the battery lifetime of the devices are limited between 3 to 5 days [47] while further optimization can extend it up to more than 20 days [48] which is reasonable for health monitoring systems if it is non-invasive system.

However, for applications such as rescue, workers safety and other critical operations discussed in this paper, the lifetime is one of the concerns, however, it is not the most important issue. On the contrary, it is the reliability of the successful transmission of packets as well as within the required latency, which are the most crucial requirements for the target WBAN-based applications discussed in this paper. Therefore, the MAC layer has to take care of these two important constraints for the specific applications explored in this paper.

#### 4.2.3. Existing State-of-the-Art on the MAC Protocols

Medium access is based on either TDMA or CSMA mechanisms. Table 8 presents a brief overview with reference to the performance of these mechanisms. Different performance metrics are presented and it can be seen that both mechanisms are effective based on the specific constraints. Most of the existing MAC protocols consider energy efficiency and delay as the prime objectives for most of the WBAN-based applications. However, in this section we will also explore more design constraints through the various studies that exist in the literature about the MAC protocols for WBAN. We have classified various MAC protocols in different categories. It includes IEEE 802.15.4-based general and WBAN-specific MAC protocols, TDMA-based low duty cycle, hybrid and cross-layer protocols, traffic-aware adaptive protocols and IEEE 802.15.6-based WBAN MAC protocols, below we will explore detailed insight of each category.

##### IEEE 802.15.4-based Protocols

IEEE 802.15.4 is a low-power, low data rate standard developed for typical wireless sensor network-based applications. Initially it also has been the main focus of researchers in the context of WBAN. Below we will present a brief survey of the IEEE 802.15.4-based WBAN MAC protocols.

The performance in terms of energy efficiency of a non-beacon IEEE 802.15.4 was investigated in [49]. The authors concluded that the non-beacon IEEE 802.15.4 results in 10 to 15 years sensor lifetime for low data rate and asymmetric WBAN traffic. However, their work considered data transmission based on periodic intervals, which is not a perfect scenario in a real WBAN. In [50], three different access schemes are evaluated through several metrics.

Considering the coexistence of contention access period (CAP) and contention free period (CFP), authors also study the mutual influences of these two traffics. The results show that the unslotted mode has better performance than the slotted one in terms of throughput and latency but with the cost of more power consumption. Further, an improved IEEE 802.15.4 MAC protocol is present in [51], with an adjustment of the beacon traffic according to packet arrival rate. The presented scheme performs better in terms of energy consumption with regards to classical IEEE 802.15.4.

Performance analysis of IEEE 802.15.4 and IEEE 802.11e for WBANs applications is presented in [52]. This paper studied the energy efficiency and QoS performance of IEEE 802.15.4 and IEEE 802.11e MAC protocols for WBANs-based applications. Authors simulated a WBAN, as well as co-existence scenarios where the body sensors operate in the presence of voice, video, and IT traffic. Their results indicate that although IEEE 802.15.4 and IEEE 802.11 e can provide an acceptable compromise between power consumption and QoS in some scenarios, there are situations (e.g., co-existence with video and heavy data traffic) in which both performance criteria cannot be met simultaneously. This highlights the need for improving existing MAC protocols or designing new solutions that can provide both extremely low power and QoS for WBANs.

Both [52,53] concluded that although 802.15.4 can provide the required QoS for BAN, however, the technology is not adaptable, scalable and power efficient to meet the requirements of various WBAN applications. It can be considered for quick and easy implementation, but the results are poor because IEEE 802.15.4 was not designed for WBANs. Therefore, a specialized MAC protocol is needed to meet all the requirements.

##### IEEE 802.15.4-based WBAN-Specific MAC Protocols

The authors in [54] proposed an energy efficient BodyMAC protocol. It uses flexible bandwidth allocation to improve node energy efficiency by reducing the possibility of packet collisions and by reducing radio transmission times, idle listening, and control packets overhead. BodyMAC is based on a Downlink and Uplink scheme in which the Contention Free Part in the Uplink sub-frame is completely collision free. Three types of bandwidth allocation mechanisms allowed for flexible and efficient data and control communications. An efficient sleep mode is introduced to reduce the idle listening duration, especially for low duty cycle nodes in the network. Simulation results show superior performance of BodyMAC compared to the IEEE 802.15.4 MAC.

Heartbeat Driven MAC protocol (H-MAC) [55], is another WBAN-specific TDMA-based protocol which originally was proposed for a star topology. The energy efficiency is improved by exploiting heartbeat rhythm information in order to synchronize the nodes. The nodes do not need to receive periodic information to perform synchronization. The heartbeat rhythm can be extracted from the sensory data and hence all the rhythms represented by peak sequences are naturally synchronized. The H-MAC protocol assigns dedicated time slots to each node to guarantee collision-free transmission. This protocol is supported by an active synchronization recovery scheme supported by two resynchronization schemes. Although H-MAC protocol reduces extra energy cost required for synchronization, it does not support sporadic events. Since the TDMA slots are dedicated and are not traffic adaptive, H-MAC protocol encounters low spectral/ bandwidth efficiency in case of low traffic.

A Medical Medium Access Control (MedMAC) protocol is proposed in [56] for energy efficient and adaptable channel access in body area networks. The MedMAC incorporates a novel synchronization mechanism in which only a multi-superframe beacon has to be listened by the nodes. An optional contention period is also available for low-grade data, emergency operation, and network initialization procedures. Initial-level simulations show that the MedMAC protocol performs better than the IEEE 802.15.4 protocol for two classes of medical applications.

##### TDMA-based Low Duty Cycle

Duty cycling is one of the most energy efficient and famous techniques for low-power devices. The energy efficient low duty cycle MAC protocol for WBANs presented in [57], is based on multi-channel and centrally controlled synchronized protocols. In this work, master-slave based network topology is used, where master is synchronized with slave based on time slots for fixed traffic. Although it achieves duty cycle in the order of 10% but the protocol is efficient only for very low static traffic. The authors in [58] proposed an energy efficient low duty cycle TDMA-based MAC protocol. It enables access to the physical layer for a hierarchical topology consisting of nodes communicating with master nodes, which in turn communicate with the monitoring station. The hierarchy removes the need for the sensors to expand power by transmitting to the monitoring station. It considered a TDMA frame of a duration of 1 s, packet size of 1250 bits with 80 bits of overhead having sampling rate of 125 samples/s. It achieves 4.51% duty cycle with each node requires 48.3 ms to completely transmit its data packet. This total time includes packet transmission, waiting time, ACK reception, and guard time.

More recently, Chen *et al.* [59] presented a statistical MAC protocol for heterogeneous-traffic human body communication with periodic synchronization for use in heterogeneous traffic networks based on human body communication (HBC). The MAC protocol is designated to ensure energy efficiency by means of flexible time slot allocation and a statistical frame. The statistical frame is intended to increase the sleep time and keep low duty cycles in each beacon period. Further, the protocol was implemented on HBC platform and the results are compared with IEEE802.15.4 (CSMA/CA), TDMA and polling protocols. Statistical MAC appeared to be the more energy-efficient, having the lowest data transfer latencies, and seems suitable to apply in heterogeneous traffic network.

In [60] the authors studied the behavior of slotted and unslotted CSMA/CA protocols and concluded that the unslotted mechanism performs better than the slotted one in terms of throughput and latency, but with a high power consumption cost.

##### Hybrid and Cross-Layer Protocols

Several hybrid mechanisms for medium access also exist in the literature. Works such as [61] have proposed a mixture of MAC techniques based on contention-based access and polling-based access and is analyzed under temporal WBAN channel models. By using hybrid techniques at different proportions, authors have evaluated how performance and energy consumption varies. The results show design trade-offs in the packet delivery versus latency versus consumed energy space. It is concluded that for the MAC design of WBANs polling-based channel access offers significant energy gains compared to contention-based access. Regarding the latency (end-to-end delay), the combination of short contention periods with long polling periods provides the most stable performance with respect to packet transmissions.

The Human Energy Harvesting (MAC) protocol (HEH-BMAC) was proposed in [62]. In this protocol hybrid polling mechanism is used which is powered by human energy harvesting for WBANs. The protocol combines two different medium access methods, namely polling (ID-polling) and probabilistic contention (PC) access, to adapt its operation to the different energy and state (active/inactive) changes that the network nodes may experience due to their random nature and the time variation of the energy harvesting sources. HEH-BMAC exploits the packet inter-arrival time and the energy harvesting rate information of each node to implement an efficient access scheme with different priority levels. In addition, the proposed protocol can be applied dynamically in realistic networks, since it is adaptive to the topology changes, allowing the insertion/removal of wireless sensor nodes. Extensive simulations have been conducted in order to evaluate the performance of the protocol and study the performance and energy tradeoffs.

A wireless EEG monitoring system is considered in the Energy-Delay-Distortion cross-layer design which is presented in [63]. In the proposed approach, transmission energy, encoding energy, application quality of service (QoS) constraints, and scheduling are jointly integrated into a cross-layer design framework. This framework is used to dynamically perform radio resource allocation for multiple users, and to effectively choose the optimal system parameters to adapt to the varying channel conditions. Furthermore, the relationship between bandwidth and energy consumption is exploited to optimally allocate time-frequency slots to the sensor nodes while minimizing the total energy consumption in the network. Further, authors have extended the traditional Rate-Distortion analysis by considering another dimension, the energy consumption, and defined the E-R-D analysis framework for EEG signal encoding and transmission under energy constraints. Using the E-R-D model, under the delay and distortion constraints, the proposed cross-layer optimization framework is able to find the best configuration of system parameters to minimize the total energy consumption. This framework jointly minimizes the total energy consumption and determines the optimal transmitted rate at physical layer, assigned time slots length, and bandwidth at MAC layer, wavelet filter length and compression ratio at application layer.

##### Traffic-Aware Adaptive MAC for WBAN

Flexible duty cycling techniques through Traffic-adaptive MAC protocol (TaMAC) is introduced in [64]. It takes into account the traffic information of the sensor nodes. The TaMAC protocol is supported by a wakeup radio that is used to accommodate emergency and on-demand events in a reliable manner. The wakeup radio uses a separate control channel along with the data channel and therefore it has considerably low power consumption requirements. Same authors also propose another traffic-based wake up mechanism that utilizes the three categories of traffic patterns of the body sensor nodes, namely: normal traffic, on-demand traffic and emergency traffic in [65]. The wakeup patterns of all body sensor nodes are organized into a table called traffic-based wakeup table.

The table is maintained and modified by a network coordinator according to the application requirements. Based on the body sensor node's wakeup patterns, the network coordinator can also calculate its own wakeup pattern. During normal traffic, both the body sensor nodes and the network coordinator send data based on the traffic–based wakeup table. The network coordinator sends a wakeup radio signal to body sensor nodes, which wake up in response to these signals, during on-demand traffic period. During emergency traffic period, the body sensor nodes send a wakeup radio signal to the network coordinator, which responds to the wakeup radio signal.

The authors in [66] present a traffic aware fuzzy-tuned dynamic delay MAC for WBAN. It exploits a fuzzy logic decision system integrating application traffic diversity to propose a dynamic controlled range for the randomly chosen back-off periods. The dynamically adjusted upper and lower bounds of back-off period introduced on top of the IEEE 802.15.4 medium access control protocol has resulted in the improvement of delivery ratio in comparison with IEEE 802.15.4. The traffic adaptive fuzzy technique has been performed on both the minimum and maximum bounds of the back-off period. The same authors later [67], proposed an improved lightweight history-based MAC Protocol for WBAN with reduced complexity by using new caching technique to consider the maximum bound of the back-off algorithm to be dynamically adjusted. The reduction in the average number of fuzzy engine runs by the proposed caching algorithm shows a significant improvement over data reliability.

TAD-MAC [4] is another traffic-aware dynamic MAC protocol for WBAN. It proposes a CSMA/CA based evolutionary and adaptive algorithm, which is based on traffic status register (TSR). Each node contains TSR, whereas the coordinator node contains a bank of TSR to compute an accurate (traffic-dependent) wake-up interval for all the body sensor nodes. The authors claim to achieve between a 3- to 6-fold increase in the lifetime with regards to other preamble sampling-based MAC protocols for various radio transceivers. Since this CSMA/CA-based protocol is able to optimize most of the energy waste such as idle listening, collisions, over hearing and overheads, it can be considered as quite effective for WBANs.

##### IEEE 802.15.6-based WBAN MAC Protocols

A review of Medium Access Mechanisms under IEEE 802.15.6 standard is presented in [68]. In this work, the MAC protocol concepts of IEEE 802.15.6 and a comparison of exiting MAC protocols (such as TMAC and IEEE 802.15.4 MAC protocol using OMNET++ with Castalia as simulating tools) of WBAN are discussed. Similarly [69] presented a brief overview of the new IEEE 802.15.6 standard. Its authors analyzed the bandwidth efficiency of the standard for CSMA/CA procedure. The efficiency results were presented for different frequency bands and data rates. They observed that increase in the payload size improves the bandwidth efficiency. Further, various proposals from different research centers and companies are presented to IEEE 802.15.6 TG6 (WBAN Task Group) in [70].

In [71] a paper on the performance of an IEEE 802.15.6 Wireless Body Area Network (WiserBAN Project) was proposed. They considered CSMA/CA algorithm which is defined in the standard, for transmitting their data via a direct link. Two different channel models for on-body communication were taken into account for comparison. Average packet loss rate (PLR), delay and throughput were evaluated through simulations. The performance achieved with the different data rates available at the 2.45 GHz band was analyzed and a comparison with a network based on IEEE 802.15.4 standard was carried out.

The results point out the impact of different channel models on the network performance, underlying the importance of the choice of the proper model for every specific scenario. Then, the comparison between the two standards showed that, for very small payloads, 802.15.4 performs better than 802.15.6 in terms of PLR, while for longer payloads 802.15.6 achieves lower PLRs. As for the delay, the values obtained for 802.15.4 are in general lower than the ones obtained for 802.15.6. Therefore, depending on the application requirements, 802.15.4 could be more suitable in some cases than 802.15.6. Anyway, in this first evaluation, for a fair comparison between the two standards, they considered only the CSMA/CA access method and they set the same user priority for all the nodes. The possibility to choose also the slotted Aloha and to set different priorities for the various applications makes IEEE 802.15.6 standard more complex, but at the same time more flexible to meet the diverse requirements of WBANs, and its performance has to be further investigated.

#### 4.2.4. Comparative Study of MAC Protocols

At the end, a comparative study of the different categories of the MAC protocols is presented in this section. Key design characteristics and performance metrics are highlighted as shown in Table 9.

On the one hand duty-cycling is necessary to reduce the energy consumption but it has to meet the dynamic traffic variations of WBANs, which becomes difficult under TDMA mechanisms that is widely used in duty cycling, however, work such as [4] could be very effective in this context. IEEE 802.15.6 MAC protocol provides higher throughput and security, greater flexibility and coexistence mechanism for applications specific designs. However, it is still to be effectively utilized and explored to have the best performance.

### 4.3. Routing Protocols

Proper routing strategy and protocols are necessary to design innovative networking functionalities to effectively handle the dynamic nature of the On-Body and Body-to-Body wireless networks. The major functionality of the networking layer is to provide a global end-to-end network connectivity between multiple WBANs that are operating in the same vicinity. This activity includes the design of innovative topology management, addressing capabilities and self-organizing Intra/Inter-WBANs routing/relaying strategies. Current routing algorithms and approaches from the field of Wireless Sensor, Mesh and *Ad hoc* Networks such as [72–75], which were primarily designed for relatively larger networks with respect to WBANs. However, the algorithms and techniques presented in them are also valid in the context of WBANs after some modifications and adjustments.

The specific applications targeted in this survey considered only non-invasive WBANs and therefore thermal radiations from the devices attached on the body and their energy efficiency (which are severe constraints for invasive WBANs) are much relaxed in the given context. However, as explained earlier in MAC protocols design attributes (*i.e.*, Section 4.2.2), it is the quality-of-service and reliability of successful transmission and reception, are most important for routing protocols as well. Below we will present the classification and state-of-the-art routing protocols for WBAN.

**Figure 17.**
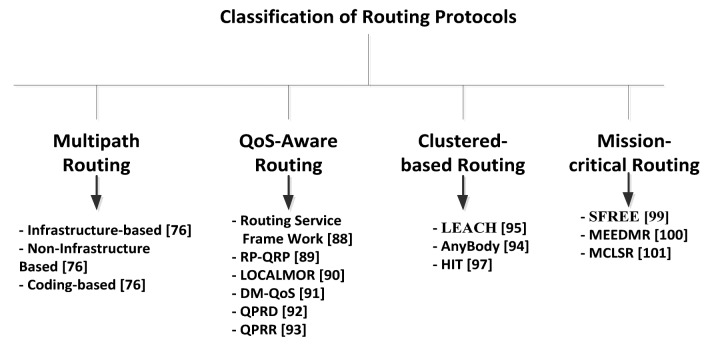
Classifications of Routing Protocols.

The classification and state-of-the-art routing protocols for WBANs can be divided into several important sub categories, which include multipath routing, QoS-aware routing, cluster-based routing, mission-critical reliable routing, and others that are shown in Figure 17, and explained in the following sections.

#### 4.3.1. Multipath Routing Protocols

Multipath routing is an efficient technique to route data in wireless sensor networks (WSNs) because it can provide reliability, security and load balance, which are especially critical in the resource constrained system such as WSNs [76]. Although there are a number of advantages associated with the multipath routing mechanism such as data reliability, data security, and energy efficiency, it also has extra overheads for the evaluation of route setup time, amount of traffic, average path length, and average delay. Multipath routing protocols can be divided into three different types of routing which include infrastructure, non-infrastructure and coding-based in order to discover multiple paths and data. The major concern of the protocols within infrastructure-based routing is to construct and maintain specific multipath infrastructure by considering location and resource capabilities. Protocols, which do not build any specific infrastructure and decide the next hop based on its local knowledge, are classified as non-infrastructure based. Whereas, when the protocols use variant kinds of coding schemes to fragment the data packet at the source node and then send the chunks through discovered multiple paths it is knows as coding-based routing approach.

In infrastructure-based multipath routing protocols, the main feature is the construction and maintenance of multiple paths from source to destination. An infrastructure provides reliable and fast data transmission because every intermediate data routing node has its next hop set up in advance. It also provides the protocol for reducing failure recovery time by assigning the alternative route, which is also discovered in advance [76]. Several techniques are exploited for reliable, secure and energy balanced designs this includes energy-aware and hierarchy-based multipath routing algorithms and are explained in much more detail in [76]. However, the focus here is to highlight multipath routing in general with reference to an overall overview of routing protocols.

In [77] a distributed, scalable and localized multipath search protocol (called energy-efficient multipath routing EEMR) to discover multiple node-disjoint paths between the sink and source nodes was proposed. Further to that, this paper also addresses a load-balancing algorithm to distribute the traffic over the multiple paths discovered. Results shows that the proposed scheme has a higher node energy efficiency, lower average delay and control overhead than other similar protocols as explained in [78]. More details on energy-aware multipath routing protocols can be found in EEAMR [79] and MRMS [80]. Infrastructure-based multipath routing can be found in [76]. In hierarchical-based multipath routing protocols nodes establishes a hierarchical relationship to discover and maintain multipath routes and because of this hierarchy it has a much reduced overheads in comparison with infrastructure based multipath routing technique presented in [76].

In [81], a unique protocol is proposed which discovers multiple paths from each node in only one route discovery process (called N-to-1 routing protocol). For path discovery, the base station periodically broadcasts the route update message and each node receiving the update message for the first time will set the sender node as parent node. This process continues recursively until the packet reaches back to the base station. This approach will lead to a breadth first spanning tree with the base station as the root of the tree.

A comparison of the main aforementioned multipath routing protocols is presented in Table 10. One of the fundamental challenges in multipath routing is how to establish the number of paths, and importantly, how to select them in an efficient way. EEMR provides high-energy efficiency by optimizing the number of paths and load balancing. However, it takes much more time in setting up various routes. EEAMR makes an attempt to reduce overheads in establishing multi paths in adaptive manner, but provides relatively lower load balancing, whereas N-to-1 protocols try to find multiple nodes disjoint paths in one route discovery process. By doing so, they optimize the energy consumption and provides higher reliability as well as security.

Details about non-structure and coding based multipath routing protocols can be found in [76]. Furthermore, the authors in [78] gave a more detailed survey of multipath efficient routing in wireless sensor networks.

#### 4.3.2. QoS-Aware Routing Protocols

Different energy efficient and QoS-aware routing protocols have been proposed for wireless sensor networks [82,83], wireless multimedia sensor networks [84,85] and MANETs [86,87], which can be used for WBANs after incorporating some customized changes according to the specific requirements of WBANs.

The Routing Service Framework [88] aims to provide user specific QoS support for WBANs. It uses a small-scale network of 20 nodes and results show that it performs well in terms of reliability and latency. However, it contains more communication and computation overhead due to control packets. Reinforcement learning based routing protocol with QoS support (RL-QRP) is proposed for biomedical sensor networks in [89]. This QoS-aware routing protocol based on geographic information and distributed Q-learning algorithm. In RL-QRP, optimal routing policies can be found through experiences and rewards without requiring any precise network state information. Simulation results show that RL-QRP performs well in terms of a number of QoS metrics and energy efficiency. Further, it fits well in the dynamic environment under various networks traffic load and mobility. Like the Routing Service Framework [88], it also uses small-scale network of 20 nodes. The packet delivery ratio decreases as the mobility level increase and the average end-to-end delay increases with the network throughput enhancement.

The authors in [90] proposed a LOCALized Multi-Objective Routing (LOCALMOR) protocol for WBANs to provide different QoS services according to the traffic type, while considering latency, reliability, residual energy, and transmission power. The protocol attempts for each packet to fulfill the required QoS metrics in a power-aware fashion. It employs memory and computation efficient estimators, and uses a multiple sink single-path approach to increase the reliability. Results show that the proposed approach performs well in terms of packet delivery ratio as well as packet delivery delay as compared to other state-of-the-art schemes.

However, all data packets are blindly disseminated towards both the primary and secondary sinks. The network traffic increases due to duplicate transmission of many data packets. This issue was partly solved in [91] (Data-centric Multiobjective (DMQoS-aware) routing protocol) by sending the data packets through single sink and it performs better in comparison with [90] by reducing latency, improving reliability and decreasing energy consumption. However, its performance decreases due to increase in the network throughput.

Based on the literature review of QoS-aware routing protocols, Table 11 provides a comparison of these protocols in terms of network size, network throughput, mobility, delay, packet delivery ratio, and energy consumption. In terms of high packet delivery and low delay, RL-QRP [89] provides best performance. Concerning priority-based routing and user specific QoS Routing Service Framework [88] achieves best performance. However, neither approach considers energy efficiency. For large-scale networks and with very low network throughput, DMQoS [91] performs better to reduce delay and improve reliability. For small-scale networks with high network throughput, QPRD [92] results into less packet delivery delay while same authors have improves the reliability of data delivery in QPRR [93].

#### 4.3.3. Cluster-based Routing Protocols

Cluster-based routing is an important class of routing protocols used in WBANs. Different methods are exploited to select a cluster head where data is transferred to the coordinator nodes through these heads to reduce the direct communication between the sensing nodes and the coordinator. The cluster head nodes are more powerful than the rest of the nodes in terms of available resources. The following sections give the overview of some of the important cluster-based routing protocols for WBANs.

Self-organization for wireless multi-hop systems can be based on reactive on-demand solutions, which are adapted to low-energy low-traffic WBANs. The authors in (AnyBody [94]) have shown that, despite the relative high cost to build and maintain a topology, a cluster-based approach is particularly suited for Body Area Networks. There are five steps of AnyBody protocol that includes: neighbor discovery, density calculation, constructing cluster head, setting up the backbone, and setting up the routing path. AnyBody uses the similar principle of Low Energy Adaptive Clustering Hierarchy (LEACH) [95], which selects the cluster head at regular time intervals to balance the energy consumption, and cluster head aggregates the data and sends to the remote station. In LEACH it is assumed that all nodes are within the range of remote base station, whereas, AnyBody addresses this problem by using a density-based cluster head selection method and using these cluster heads to build a backbone network. The main advantage of AnyBody [94] is that the number of clusters remains almost constant even when the number of nodes in the network are increased, whereas, in LEACH as the number of nodes increases, the number of clusters has to increase as well. This results in a bigger cluster size of AnyBody as compared to LEACH. Furthermore, AnyBody also reduces the cost of setting up the cluster.

Tree based algorithms have been used in solving many computational complex problems. Using this structure, the authors in [96] proposed a secure cluster-based multipath routing protocol for multimedia content. The multimedia (which is the essential for the specific critical and rescue applications) requires a better QoS and in order to achieve that, the nodes in the network have to process the data frequently and which will causes faster depletion of energy at nodes. To achieve the data reliability and to provide a faster data computation, the protocol uses a hierarchical structure of multiple paths based on clusters. The multiple paths are discovered by message broadcasting and each cluster head node is connected to the sink either directly or via other cluster head node.

Hybrid Indirect Transmission (HIT) [97] is another cluster-based data gathering hybrid architecture protocol. HIT utilizes parallel processing to minimize energy consumption and network delay for both inter-clustered and intra clustered communication. Results show that it performs better (both in terms of energy and delay) when it is compared with Low Energy Adaptive Clustering Hierarchy (LEACH) [95] and Power Efficient Gathering for Sensor Information System (PEGASIS) [98] under direct transmission for small number of nodes, however it consumes more energy in dense networks.

PEGASIS significantly reduces the overhead of dynamic cluster formation by providing an opportunity to all the nodes (turns-by-turn) to transmit the fused data to the sink node and as a result to improve the network lifetime. It considered variable (small-to-medium) size network and optimize the sole metric, *i.e.*, global energy consumption. However, it does not consider other performance metrics to analyze the complete performance.

HIT results in low packet delivery delay and energy consumption and considerable packet delivery ratio. It also considered security as additional metric and provides high throughput. On the other hand, AnyBody improves significantly the packet delivery ratio for large network. It consumes more energy and it does not consider the packet delivery latency. As shown in Table 12, cluster-based routing protocols for WBANs are compared in terms of delay, packet delivery ratio, security, and energy consumption performance metrics.

#### 4.3.4. Mission-Critical Routing Protocols

Mission-critical applications over wireless sensor networks (WSNs) need to support fast, reliable, and fault tolerance in its routing protocol [99]. In this context, the Secure, Fast Rebuilding, and Energy Efficient (SFREE) Routing Protocol for Mission-Critical Application over WSNs is presented in [99]. The presented protocol is compared to LEACH and Hierarchical Periodic, Event-driven and Query-based (HPEQ) protocols, and it is reported that the proposed protocol provides reliability with reducing processing time and energy dissipation through cluster based authentication mechanism and delayed propagation of management messages. It is shown through simulations that (10 to 15) % of overall energy dissipation is reduced along with 40% reduction of cluster rebuilding time. However, the protocol is designed for static topology and the results will deviate under network mobility.

Tree structures can also used in mission critical applications, for example, the authors in [100] propose a paper on minimizing end-to-end delay using a multipath routing (MEEDMR) protocol. The multiple paths and alternate paths are discovered by using tree based search algorithms from source to destination considering packet loss and packet delay. To provide reliable transmission, the data packets are distributed throughout multiple paths. Whenever the end-to-end reliability falls below a certain value, the route maintenance is performed.

In [101] its authors introduced a link-state routing protocol for multi-channel multi-interface wireless networks with an objective to minimize the broadcast overheads. For this purpose cluster-heads have been used and the proposed multi-channel link-state routing (MCLSR) protocol is implemented on a test bed which is based on Linux 2.4.6 Kernel, where two radio interfaces, are embedded in the kernel, as kernel modules. Authors have compared performance of their proposed protocol with an ad-hoc on demand distance vector routing reactive protocol [102], which is also tailored for multi-channel multi interface networks. The measurements on our test bed show that the proposed link-state routing protocol provides transient route discovery time and lower packet drop rate, which is important in mission-critical networking

The authors in [103] specifically focus on interoperability between heterogeneous networks. They address the routing problem in scenarios where nodes that belong to one organization (or network) could transport traffic to nodes from another organization (or network). This is crucial for maintaining survivability and efficiency in a harsh environment. The particular interest is that, how much benefit and overhead can be achieved when different organizations collaborate to support critical applications without violating organization induced constraints. In this work, a new metric is defined to model different levels in an organization-aware network using hierarchical addresses, and organizationally shortest path (OSP). Also, the proposed metric is backward compatible with other famous protocols such as optimized link state routing (OLSR) protocol. Simulation results demonstrate that the proposed method provides survivability and efficiency in battlefield environment while keeping the cross-organization data transfer at a low level.

To meet the stringent requirement of reliably transmitting data for critical disaster evacuation and military applications, the authors in [104] propose a lightweight and energy-efficient joint mechanism for packet loss recovery and route quality awareness in WSNs. In this protocol, overhearing feature for characterizing the wireless channels as an implicit acknowledgment (ACK) mechanism is used as proposed in [105]. In addition, the protocol allows for an adaptive selection of the routing path, based on a collective cooperation within neighborhood. After a detailed survey on WBAN communication stacks, in the next section, open issues, challenges, and opportunities are explored and discussed.

## 5. Open Research Issues, Challenges and Opportunities

Research on Wearable WBAN systems started significantly, worldwide, a decade ago, and encompasses a wide range of multidisciplinary and cross-sectorial research topics, including on-body propagation and antennas, medium access control (MAC), networking, security, and so on.

In the past, many ambitious research projects (as shown in Table 13) have been devoted to the design of efficient WBANs solutions, such as CodeBlue [106], ASNET [107], HealthService24 [108], AID-N [109], MIMOSA [110], SMART [111], CareNet [112], WiMoCA [113], BANET [114], ProFiTex [115], and MobiHealth [116]. More recently, new research projects have started. Some notable examples are WearABAN [117], WiserBAN [118], WearIT@Work [119], ALARP [120] and more recently CORMORAN [121], CAALYX-MV [122] , Help4Mood [123] and suWBAN [124].

The proposed WBAN solutions target a wide range of applications, including ambient intelligence, healthcare monitoring, disaster aid network, sport, gesture detection, *etc*. However, as shown in Table 13, most of the proposed short-term Wearable WBAN solutions are generally based on existing communications standards such as PANs (e.g., Bluetooth), WSNs (e.g., Zigbee), MANETs (e.g., WiFi, Multi-hop IEEE 802.11), cellular, *etc*., and thus, do not meet the specific requirements and constraints of WBANs as already discussed in Section 2.

Moreover, most of these projects are targeting closed or independent WBAN systems, which can only communicate with the outside world via pre-existing cellular, WiFi or static WSN network infrastructures, and with a specific focus on medical related applications.

There have been so far only a few research studies related to the issue of self-organizing, cooperative and multi-hop communications between multiple WBANs (*i.e.*, hybrid On-Body and Body-to-Body Communications) in infrastructure-less environments. All these major research issues have yet to be resolved in order to enable the emergence of future ubiquitous communication and monitoring systems enabling life-critical and rescue operations, public safety and preparedness.

In the following sections, the major design challenges, open research issues, and opportunities are discussed in details using a top-down approach, from applications down to the lower layers.

### 5.1. WBAN Design Challenges for Life and Safety Critical Applications

The specific context of life and safety critical applications introduce several new design constraints and research challenges in comparison to general-purpose or medical WBAN solutions, as shown in Table 14. Indeed, classical WBAN solutions are essentially designed as closed systems, which are eventually open to the outside world via pre-existing network infrastructures. More specifically, life and safety critical related application scenarios are characterized by the following main differences in comparison to general-purpose or medical WBAN solutions.

First, mobile workforces (e.g., rescue teams, workers, *etc*.) are generally *deployed in harsh or disaster environments* and require effective communication, coordination and monitoring capabilities: (i) to ensure their safety in presence of unhealthy environments or hazards ; (ii) to optimize their tasks on operational theaters; and (iii) to effectively communicate and transmit real-time information to their command center for proper decisions making and workforces management.

Second, during practical cases of deployment in harsh or disaster environments: (i) *broadband or local network infrastructures* (e.g., 4G, 3G, LTE, WiFi, *etc*.) *might be absent* (e.g., desert, offshore oil and gas fields), *damaged* (e.g., in case of major fire) *or overloaded* (e.g., in high density area such as stadiums or shopping malls); (ii) mobile workforces might face various *uncertainties and hazards* (e.g., low visibility, high temperature, emanation of toxic gases, major fire, no a-priori knowledge of the building indoor layout, *etc*.); and (iii) Sensitive and/or real-time information should still be transmitted from the deployed mobile workforces teams to external command centers to ensure timely and effective decisions making.

To that end, each team's member will be fitted with a set of—physiological, environmental, communication and kinematics—Wearable Wireless Sensor Devices. On the first hand, short-range and multi-hop *on-body communications* will be required to effectively collect and transmit all collected sensors readings at the WBANs coordinator (e.g., PDA). On the other hand, medium-range, cooperative and multi-hop *body-to-body communications* will be required to: (i) ensure the end-to-end network connectivity with external command centers; (ii) route sensitive and real-time information to these centers; and (iii) improve the communication reliability in case of harsh propagation conditions and severe links obstructions.

In this context, the resulting Wearable WBAN systems for safety and life critical applications should be autonomous, self-organizing, large scale, robust to external interferences, energy efficient, secure, support various data types and rates (e.g., voice, data, real-time traffic, periodic traffic, urgent alarms and events, *etc*.). Moreover, they should enable cooperative (or collaborative), reliable and real-time communications while providing various quality of services (QoS) and security levels. In order to better understand the requirements of life and safety critical applications, a specific use case related to worker safety in a harsh environment is considered.

#### Workers Safety in Harsh Environment

Specific details about workers safety in a harsh environment as an application scenario is already provided in Section 2.2.2. In such an application context, WBAN systems should support various monitored data types and sensors. As shown in Table 15, the monitoring requirements for the workers safety scenario can be classified into three main categories. The first category deals with normal and daily worker health monitoring and protection in oil and gas fields and refineries, petrochemical and mining industries as well as roadside and building workers. The second category adds specific requirements concerning movement and activity monitoring, whereas the third category is about protection and safety from hazards and poison gasses, fall detection as well as detection of necessary safety equipment.

In order to better understand the specific data requirements of the sensors in worker safety scenarios a brief analysis is presented as follows. First for the health monitoring, most of the collected vital sign information such as temperature, oxygen saturation, heart rate, pulse and respiratory count can be transmitted to the WBAN coordinator just in one packet/s (for example, since data packet at the physical layer in IEEE 802.15.6 can be up to 256 Bytes and as the information in these sensors are not more than few bytes). Therefore, information can also be aggregated and finally transmitted after one second.

However, the data requirements for ECG, EMG, and EEG signals are much more in comparison with the above basic data, therefore, these signals are often compressed to lower rates. For example, ECG can be compressed up to 4 Kb/s/electrodes (with a sampling rate of 250 Hz and 16 bits resolutions [125]), which means 12 data packets per second for three electrodes (which is a minimum requirement for a physician to understand complete cardio activity) are required. However, the effective number of packets will be more after adding the lower layers overheads). For the case of EEG, data can be compressed up to 40 Kb/s/electrodes [126], which will result in 40 data packets per second for each electrode. Similarly more than 50 packets for EMG could be required [127].

Second, for the movement activity and protection cases, accelerometers', gyroscopes' and gas sensors' data requirements can be met in one-to-two packets/s. Overall, the MAC super frame has to manage more than 100 packets/s of 256 Bytes which is more than 200 Kb/s (only for the payload).

From the above analysis, it can be seen that the low-power and high data rate requirements of the safety and critical applications can only be properly realized and meet by using the WBAN-specific IEEE 802.15.6 standard. It can also be seen that the packet delay has to be managed properly (for example 125 ms is the maximum latency for the case of IEEE 802.15.6). Due to critical nature of the applications as well as due to limited memory size of the sensor devices as it is not possible to keep buffering the packets more than few seconds, therefore, the time constraint could be very crucial for these new applications.

### 5.2. Communication and Networking Protocols Challenges

Existing MAC protocols, which claim to be effective for WBANs, are not completely compliant with the key attributes and characteristics described in Section 4.2.2. Furthermore, the MAC layer of the IEEE 802.15.6 standard [9] needs to be maximally exploited to meet the required design constraints. MAC layer should be robust enough to support multiple WBANs reliably and has to be energy efficient. Moreover, QoS requirements for transmission of data as soon as possible need to be handled in appropriate manner. In order to achieve many design objective through proposed IEEE 802.15.6 MAC protocol is still to be investigated and is an open challenge as discussed above in the previous section.

With regards to effective networking for WBANs, appropriate mobility modeling is very important. Since the human body is constantly moving, sensor nodes on or inside the body have mobility features. With the body movements, the distance between sensor nodes will be constantly changing. This will cause serious topological partition problems in that some links between nodes are broken and built frequently. This kind of network is called Delay Tolerant Network (DTN), whose routing protocol is very difficult to design and realize [89]. Existing approaches in the Mobile *Ad hoc* [128] or the Delay Tolerant Networking (DTN) [129] fields need to be evaluated and coupled with Wearable WBANs. For example, [130] deals with a limited multi-hop WBAN and proposes an epidemic routing strategy coupled with a TDMA MAC scheduling over a single BAN.

For low-range communication, an opportunistic and on-demand routing scheme coupled with service discovery may be sufficient to enable collaboration across WBANs. Regarding long-range communications, work such as WASP and CICADA [131] use cross-layer data-forwarding schemes to extend the structure of the WBAN in a tree-like topology. However, depending on the considered applications, the WBANs' communication architecture cannot always be organized around a central WBAN coordinator. In this context, a promising research trend is the development of opportunistic and mobility aware communication stack.

Last but not least, energy efficiency is a paramount issue to guarantee that WBAN systems can operate continuously for up to several days between batteries recharge cycles. Energy consumption can be divided into three categories, *i.e.*, radio communication, data processing, and sensing. Hence, special attention should be paid on designing energy efficient WBAN communication stacks where available radio communications and data processing at the WBANs' nodes are efficiently managed. In this context, promising research trends concern the development of low control overhead, low delay transmission, and cross-layer MAC and Networking layers. Finally, as will be discussed further in Section 5.4, the proper management of interference is also extremely important to improve energy efficiency and enable the emergence of green WBANs.

### 5.3. Security and Privacy Challenges

Given the specific requirements and constraints of wearable WBAN solutions, in terms of limited resource capabilities and harsh environmental conditions, the design of new security and privacy mechanisms remains an important challenge yet to be achieved. Unlike traditional communication networks, there has been relatively little research so far on solving the security and privacy issues in Wearable WBANs [132–134]. The major security and privacy requirements for Wearable WBAN solutions can be classified in three main categories [132]:

- The *data storage security requirements* in terms of data confidentiality, data integrity assurance and data dependability;
- The *data access security requirements* in terms of users' access control, users' accountability, users and/or WBANs nodes revocability and data non-repudiation;
- The *data communication security requirements* in terms of users and/or WBANs nodes authentication, WBANs systems availability, data confidentiality, data integrity, and data freshness.

As already discussed in Section 3.6, the IEEE 802.15.6 standard [9] is already proposing three basic security levels for WBAN systems, including the *unsecured communications* (level 0), the *authenticated but not encrypted communications* (level 1), and the *authenticated and encrypted communications* (level 2). In this latter security level, all the messages are transmitted within authenticated and encrypted frames, enabling thus the authenticity, integrity, confidentiality, privacy and replay defense of the exchanged messages. However, these traditional encryption and authentication mechanisms remain not perfectly suitable for WBAN systems due to their stringent requirements in terms of limited power consumption, computation capability, heterogeneous Intra/Inter/Beyond-WBANs communications, infrastructure-less deployments, and so on.

In this context, new lightweight security and privacy paradigms are currently being developed, such as the efficient generation and management of cryptographic keys using biometric techniques [135] (*i.e.*, based on collected physiological signals, such as ECG, *etc*.) and physical channel based approaches [136] (*i.e.*, based on radio channel impulse responses and/or RSSI values). Another promising research trend is the development of new schemes to solve WBANs' dependability issues [134], defined as the conjunction of reliability, security, and availability, from both an Intra-WBAN and Inter-WBAN communication perspective.

### 5.4. Coexistence and Interoperability Challenges

Neighbouring WBANs operating in the same frequency bands are likely to interfere with each other. Coexistence and interoperability between co-located WBANs are thus a key requirement to enable the emergence of practical and effective Wearable WBAN systems. Indeed, Inter-WBAN interference is expected to seriously limit the performances of WBAN communications.

A WBAN coexistence algorithm should ensure a proper functionality of multiple co-located WBANs (*i.e.*, on the same body, or in the same environment) and carry out, either independently or cooperatively, their communications without severe performance degradation. Several WBAN coexistence schemes have been proposed in the literature (e.g., [137]), as well as by the IEEE 802.15.6 standard [9], as already discussed in Section 3.5, including the beacon shifting, channel hopping and interleaving mechanisms. However, it should be noted that most of these coexistence schemes are *non-collaborative*, where Inter-WBAN interference is mitigated as much as possible to maximize the throughput of a single WBAN. In other words, the IEEE 802.15.6 was mainly designed to enable efficient Intra-WBAN and Beyond-WBAN communications (*cf*. Figure 1), whereas Inter-WBAN communications are not supported efficiently.

The design of efficient Wearable WBAN systems for the context of life and safety critical applications (*cf*. Table 14), will require the support of *cooperative* and *collaborative* coexistence mechanisms between co-located WBANs, where coordinators and/or on-body sensors of different WBANs can communicate with each other. Existing collaborative and non-collaborative coexistence approaches, which were initially presented in [138] for PANs, could be enhanced and improved to meet the specific requirements of WBANs, especially to enable efficient Inter-WBAN communications.

### 5.5. Mobility and Channel Modeling Challenges

The modeling of the on-body radio channel has attracted more attention in the research community, however, only a few studies have addressed the issue of the time-variant channel model. The human body mobility should be taken into account to enable the design of efficient cooperative communication protocols for WBANs applications. Moreover, the characterization of these phenomena is difficult in terms of channel sounding. This is why VNAs (VNA: Vector Network Analyzer.) are often exploited to characterize the static WBAN channel by means of frequency domain measurements [139].

From IEEE 802.15 TG6 only few studies have targeted the characterization of the dynamic on-body channel (namely CM3 in [140]). For instance, NICT have performed measurements using a channel sounder at 4.5 GHz [141], while NICTA characterized the on-body narrowband channel on two ISM (ISM: The industrial, scientific and medical (ISM) radio bands.) frequency bands: 820 MHz and 2.36 GHz [142]. The high dispersion of results presented in literature, even in the same channel model; e.g., [140], is due to the fact that the proposed distance-dependent path-loss models are inconsistent for on-body channels [143,144].

In future, works such as [145] are needed further to investigate the impact of realistic WBAN radio channel and mobility conditions on the network topology dynamics and the performance of network and routing architectures. Similarly, biomechanical cylinder-based channel models, such as [38], are important to generate realistic mobility patterns as a function of time.

With respect to communication protocols, for example, to evaluate Intra-WBAN communication performance, a time varying channel model is necessary. In particular, temporal and spatial correlation and associated features are important to accurate model the mobile WBAN channel. Whereas, for inter WBAN ‘body-to-body’ channel variations have to be considered which are much more mobility and environment dependent. In such cases, specific mobility models such as group mobility of rescue team or mobile workforce are necessary to be validated first, and then to evaluate their impact on the performance of the body-to-body communications.

Concerning the worker safety scenario discussed from the application perspective in Section 5.1, Table 16, presents different sensors and their possible locations on the human body in various body postures along with corresponding movement of individual sensor. Four different body positions (*i.e.*, sitting, standing, walking, and running) can be envisioned for a typical working environment. The positioning of the sensors and devices is important, first, it should be comfortable to the worker; second, the corresponding links between each sensor should be reliable. From past different studies and experiences (as explained in Section 4.1.2), positions presented in Table 16 are considered as one of the examples, though real experiments are necessary to validate them. Finally, mobility column reflects individual sensors mobility in different body positions.

In order to design or select appropriate channel model and mobility patterns, radio link between each sensor is necessary to identify. In this regard, cross-radio-links (between sensors) under workers safety scenario for sitting, standing, walking and running positions can be analyzed for accurate modeling. For the given application, three types of links can be considered which are, line-of-sight (LOS), non-line-of-sight (NLOS) and periodic LOS/NLOS (P-LOS/NLOS).

### 5.6. WBANs Performance Evaluation, Simulation and Experimentation Challenges

Due to the high complexity of theoretical models and the unavailability of commercial IEEE 802.15.6 standard-compliant devices, simulation is currently considered as the most convenient methodology to evaluate the performance of Wearable WBANs and their protocol stacks.

However, none of the existing simulation environments takes accurately into account the specific characteristics of WBANs, such as the physical layer (e.g., time-variant channel, temporal and spatial correlations of radio losses, body shadowing effects, *etc*.) and the mobility behavior of humans (e.g., biomechanical body, group/social mobility behavior). Most research studies related to on-body and body-to-body communication networks are still based on unrealistic simulation parameters and physical models.

Based on experimental channel measurements, abstracted radio link models as well as realistic Intra-WBAN and Inter-WBAN mobility models, existing simulation environments should be enhanced. The ideal WBAN simulator should include a realistic abstraction of the Inter-body channel variations under mobility for the lower layers and realistic higher layers, hence easing the design and evaluation of new MAC and networking functionalities. In this context, one shall carefully mind the challenges resulting from the integration of different models into one single tool (e.g., in terms of interfaces, overall consistency, heterogeneous levels of abstraction, computational cost, *etc*.). In particular, the best compromises between physical realism and computational tractability, as required by cooperative Inter-WBAN scenarios, should be determined in the future.

## 6. Conclusions and Future Works

In this paper an application-specific survey for WBANs wa presented. Currently, rescue and critical missions, mobile workforce safety and management-based applications are not dealt with through wearable technology. However, due to tremendous capabilities and emergence of smart and tiny wearable technologies in near future it will be possible to have effective measure in harsh and disaster environments. In this context, it is necessary to understand the capabilities and limitations of the existing approaches and standards.

This survey highlights the important features and requirements of the new potential applications for WBANs. It emphasizes on the utilization of the new features introduced in key enabling WBAN-specific standard, *i.e.*, IEEE 802.15.6, for the given applications. A comprehensive and comparative study of protocol stack design for WBANs is carried out. It is evident from the current research that existing algorithms and protocols for physical, MAC and routing cannot completely satisfy the specific requirements emerged from the new applications paradigm discussed in this paper.

Several limitations of the existing research projects are highlighted and further it is mentioned that the lack of IEEE 802.15.6-based radio transceivers and devices are limiting the penetration for the significant and realistic research and applications development. Finally, several open research issues and challenges are discussed and explored as possible topics for future work.

## Figures and Tables

**Table 1.** IEEE 802.15.6 WBAN Requirements [24].

	**WBAN Functional Requirements**
Topology	One-hop star, two-hop star, bidirectional link
Setup Time	Insertion/removal <3 s
Devices Number	Typically 6, up to 256
Data Rate	10 Kb/s–10 Mb/s
Range	≈3 m with low data rate under IEEE Channel Mode
PER	<10% with a link success probability of 95% over all channel conditions
Latency	<125 ms (medical); <250 ms (non-medical)
Jitter	<50 ms
Reliability	<1 s for alarm; <10 ms for applications with feedback
Power Consumption	>1 year (1% LDC + 500 mAh battery); >9 h (always ON + 50 mAh battery)
Intra-Coexistence	10 WBANs in a volume of 6 × 6 × 6 m
Inter-Coexistence	Environment (WiFi, Bluetooth, *etc*.)

**Table 2.** Typical WBAN Sensors and Data Rates.

**WBANs Applications**	**Signals**	**Data Range**	**Frequency (Hz)**	**Accuracy (bits)**	**Data Rate**
Medical/Health	Glucose Concentration	0–20 mM	40	12	480 bps
	Blood Flow	1–300 ml/s	40	12	480 bps
	ECG	0.5–4 mV	500	12	6 Kbps
	Respiratory Rate	2–50 breaths/min	20	12	240 bps
	Pulse Rate	0–150 BPM	4	12	48 bps
	Blood Pressure	10–400 mm Hg	100	12	1.2 Kbps
	Blood pH	6.8–7.8 pH	4	12	48 bps
	Body Temperature	32–40 °C	0.2	12	2.4 bps

Non-Medical	High Quality Audio	-	-	-	1.4 Mbps
	Voice	-	-	-	100 kbps
	Video	-	-	-	1–2 Mbps
	GPS positions	-	1	32	96 bps
	Motion Sensor	-	100	16	4.8 Kbps

**Table 3.** WBAN-Related Standards.

	**IEEE 802.11 a/b/g/n (WiFi) [26]**	**IEEE 802.15.1 (Bluetooth) [27]**	**IEEE 802.15.4 (Zigbee) [28]**	**IEEE 802.15.4 a (UWB) [29]**	**IEEE 802.15.6 (WBANs Standard) [9]**
Modes of Operation	*Ad hoc*, Infrastructure	*Ad hoc*	*Ad hoc*	*Ad hoc*	*Ad hoc*
Physical (PHY) Layers	Narrowband	Narrowband	Narrowband	Ultra Wideband (UWB)	Narrowband, Ultra Wideband (UWB), Human Body Communication (HBC)
Radio Frequencies (MHz)	2400, 5000	2400	868/915, 2400	75–724, 3128–4859, 3000–5000, 6000–10000	402–405, 420–450, 863–870, 902–928, 950–956, 2360–2400, 2400–2438.5
Power Consumption	High (≈800 mW)	Medium (≈100 mW)	Low (≈50 mW)	Low (<50 mW)	Ultra low (≈1 mW at 1 m distance)
Maximal Signal Rate	Up to 150 Mb/s	Up to 1 Mb/s	Up to 250 Kb/s	Up to 27.24 Mb/s	10 Kb/s to 10 Mb/s
Communication Range	Up to 250 m (802.11 n)	100 m (class 1 device)	Up to 75 m	Up to 30 m	Up to 10 m (nominal ∼2 m)
Networking Topology	Infrastructure based	Ad-hoc very small networks	Ad-hoc, Peer-to-Peer, Star, Mesh	Ad-hoc, Peer-to-Peer, Star, Mesh	Intra-WBAN: coordinated, uncoordinated, 1/2-hop star. Inter-WBANs: non-standardized
Topology size	2007 devices for structured WiFi BSS	Up to 8 devices per Piconet	Up to 65536 devices per network	Up to 65536 devices per network	Up to 256 devices per body, and up to 10 WBANs in a volume of 6 × 6 × 6 m
Target Applications	Optimized for Data Networks	Optimized for Voice Links	Optimized for sensor, home automation, *etc*.	Optimized for short range and high data rates, localization, *etc*.	Health Monitoring, Sports, Disability Assistance, Body Centric application, *etc*.

**Table 4.** Scenarios and description of the IEEE 802.15.6 channel models [31].

**Scenario**	**Description**	**Frequency Band**	**Channel Model**
S1	Implant to Implant	402–405 MHz	CM1
S2	Implant to Body Surface	402–405 MHz	CM2
S3	Implant to External	402–405 MHz	CM2
S4	Body Surface to Body Surface (LOS)	(13.5, 50, 400, 600, 900) MHz (2.4, 3.1–10.6) GHz	CM3
S5	Body Surface to Body Surface (NLOS)	(13.5, 50, 400, 600, 900) MHz (2.4, 3.1–10.6) GHz	CM3
S6	Body Surface to External (LOS)	(13.5, 50, 400, 600, 900) MHz (2.4, 3.1–10.6) GHz	CM4
S7	Body Surface to External (NLOS)	(13.5, 50, 400, 600, 900) MHz (2.4, 3.1–10.6) GHz	CM4

**Table 5.** Proposed Channel Model for NB [34].

	**Hospital Room**	**Anechoic Chamber**
Path-Loss Model and Parameters	*PL*(*d*) = *a*.*log*_10_(*d*) + *b* + *N*	
a	6.6	29.3
b	36.1	16.8
*σ_N_*	3.80	6.89

**Table 6.** Small-scale fading using Ricean distribution [31].

**Small-Scale Fading**	***K*_dB_** **= *K*_0_** **−** ***m*_K_** ***P*_dB_+*σ*_K_*n*_K_**
*K*_0_ [*dB*]	30.6
*m*_K_ [*dB*]	0.43
*σ*_K_ [*dB*]	3.4
Parameters of the mean value of the delay spread	
Distance [cm]	t_rms_ [ns]
15	6
45	16
Parameters of the 90% cumulative value of the delay spread	
Distance [cm]	t_rms_ [ns]
15	11
45	22

**Table 7.** Comparative analysis of existing channel models.

**Model Descriptions**	**Scenarios**	**Path Loss**	**Propagation Effects**	**Link Conditions**	**Link Type**	**Mobility**
Simulations-based space-time dependent channel model [38]	Intra-WBAN. Indoor or Anechoic Chamber	Combination of frequency, distances in free space and around the body	Spatial and temporal characteristics based fading. Shadowing due to body parts length and size.	LOS/NLOS	Hip to Wrist/Foot/ Thigh. Arm to Foot and Head to Head	Standing. walking and running
Measurement-based time-varying model [39]	Intra-WBAN. indoor or Anechoic Chamber	Time-frequency and scenario-based	Slow and fast fading. Shadowing correlation between links.	LOS/NLOS	Hip to Chest/right thigh/ right wrist/ right foot. *etc*.	Standing still, walking and running on the spot
Measurement and periodic characteristics-based model [41]	Intra-WBAN. indoor or anechoic	Distance and periodic function	Slow and fast fading along with Periodic Correlation	LOS/NLOS, Periodic LOS and Periodic NLOS	Hip to Ankle/Wrist, Wrist to Wrist/Chest, Chest to Wrist/Hip	Standing, walking and running
Simulation-based On and Off Body Multi antenna-channel model [37]	Intra-WBAN. Indoor	Geometrical-based statistical model	Multipath cluster of scatters	LOS/NLOS	Head to Front/Back	Walking
IEEE proposed models [31])	Intra-WBAN. indoor or anechoic	Distance-based	No spatial or temporal features considered.	LOS/NLOS	Around torso and on-front part o the body	Static

**Table 8.** Comparison of medium access mechanisms.

**Performance Metrics**	**CSMA/CA**	**TDMA**
Energy Efficiency	Low to medium	High
Synchronization	Not Required	Needed
Bandwidth Utilization	Low	High
Scalability	High	N/A
Reliability	Low to Medium	High
QoS	Low	High

**Table 9.** Summary on existing major WBANs MAC protocols.

**MAC Protocols**		**Key Characteristics**	**Performance Metrics**
802.15.4-based	Changle [50]	Three access schemes are considered with multiple traffic and coexistence capabilities.	Better throughput and reduced delay but higher power consumption.
	Ullah [51]	To optimize wake-up schedule of 802.15.4 non-beacon mode.	Improved energy efficiency and delay with reference to standard 802.15.4.
Low Duty Cycle and Traffic-Aware	Omeni [57]	Multichannel and centrally controlled (master-slave) synchronization for fixed traffic.	It requires high synchronization overheads and not suitable for dynamic traffic because of higher packet lost.
	Markovic [58]	Collision avoidance having low synchronization overheads.	Energy efficient with 4.5% duty cycle but higher latency.
	Alam [4]	It uses traffic-status-register-bank to keep the track of traffic variations through an evolutionary adaptive algorithm.	Traffic-adaptive energy and latency efficient, scalable and flexible but sensitive to abrupt and burst traffic.
WBAN-Specific	Heartbeat [55]	Uses heartbeat rhythm instead of periodic synchronization beacons for time synchronization.	Low synchronization overheads and optimized collisions energy efficient, but higher latency, lower throughput and non-adaptive to traffic variations.
	MedMAC [56]	Maximize energy efficiency through dynamic adjustments for QoS requirements	Energy and latency efficient for low-to-medium traffic patterns.
	BodyMAC [54]	Introduced efficient downlink and uplink with effective and flexible utilization of bandwidth	Optimizes energy efficiency by reducing idle listening, collisions and overheads
802.15.6-based	802.15.6 MAC [9]	It provides higher throughput and security, greater flexibility (both TDMA and CSMA/CA as well as hybrid medium access) and applications specific control	High throughput and data rates, better coexistence and security techniques.

**Table 10.** Comparison of the Performance of Multipath Routing Protocols.

Protocols	Objective	Performance Metrics					
		**Energy Efficiency**	**Load Balancing**	**No. of Paths**	**Delay**	**PDR**	**Route Set up Time**
EEMR [77]	To discover node-disjoint paths and properly distribute the load	Very High	High	Low	Low	High	Medium
EEAMR [79]	To Achieve energy efficiency through adaptive low overhead routing	Very High	Medium	Low	Low	High	Medium
MRMS [80]	To Achieve energy efficiency	Very High	High	Low	Low	Very High	Low
N-to-1 [81]	To Create one route discovery for each node	High	Low	Low	Medium	High	Medium

**Table 11.** Comparison of QoS-Aware Routing Protocols.

Protocols	Objective	Performance Metrics					
		**Network Size**	**Network Throughput**	**Mobility**	**Delay**	**PDR**	**Energy Consumption**
Routing Service FW [88]	To provide priority-based routing with user specific QoS	Small	N/A	Yes	N/A	Medium	N/A
RL-QRP [89]	To Achieve high packet delivery ratio and low delay	Small	Low	Yes	High	High	N/A
LOCAL MOR [90]	To provide Data dependent QoS	Medium	N/A	Yes	Low	High	High
DMQoS [91]	Data-dependent QoS	Large	Very Low	Yes	Low	High	Medium
QPRD [92]	To reduce end-to-end delay	Very Small	High	Yes	Very Low	High	Low
QPRR [93]	To improve end-to-end reliability	Small	High	Yes	N/A	High	Low

**Table 12.** Comparison of Clustered-based Routing Protocols.

Protocols	Objective	Performance Metrics					
		**Network Size**	**Network Throughput**	**Security**	**Delay**	**PDR**	**Energy Consumption**
HIT [97]	To maximize lifetime by reducing direct transmission to sink	Large	High	Yes	Low	Medium	Low
AnyBody [94]	To achieve high packet delivery ratio and low delay	Large	High	No	N/A	Very High	High
PEGASIS [98]	To reduce the energy consumption in data-gathering networks	Small to medium	High	No	N/A	Medium	Low

**Table 13.** Comparative Study on Existing WBANs Projects.

	**Projects**	**On-Body Communications**	**Body-to-Body Communications**	**Off-Body Communications**	**Target Applications**
Before 2010	CodeBlue (2005) [106]	Wired	Zigbee (Mesh)	N/A	Medical Care

	ASNET (2006) [107]	Wired or WiFi	Wired or WiFi	Internet/GSM	Remote Health Monitoring

	HealthService24 (2006) [108]	Wired	UMTS/GPRS	UMTS/GPRS/ Internet	Mobile Healthcare

	AID-N (2007) [109]	Wired	Zigbee (Mesh)	Wifi/Cellular/ Internet	Mass Casualty Incident

	MIMOSA (2008) [110]	RFID/Bluetooth/Wibree	UMTS/GPRS	Internet	Ambient Intelligence

	SMART (2008) [111]	Wired	WiFi	N/A	Health Monitoring in Waiting Room

	CareNet (2008) [112]	N/A	Zigbee	Internet/Multihop WiFi	Remote Health Monitoring

	WiMoCa (2008) [113]	Wireless (Star Topology based MAC)	Bluetooth	WiFi/Internet/ Cellular	Sport/Gesture Detection

	BANET (2008) [114]	Star/Mesh (Bluetooth LE)	Coexistence only	N/A	Medical, Health and Sport

	ProFiTex (2008) [115]	Wired	N/A	Wired	Safety and efficiency of fire fighters

	MobiHealth (2009) [116]	Manually	Zigbee/Bluetooth	GPRS/UMTS	Ambulatory Patient Monitoring

2010 - Present	WearABAN (2010) [117]	Star mesh hybrid topology (868MHz)	N/A	PC(WiFi/Cellular/ Internet)	Daily life, Game and rehabilitation
	WiserBAN (2010) [118]	Star/Mesh (IEEE802.15.4 or Bluetooth)	N/A	Smartphone (Internet/Cellular/ WiFi)	Medical
	WearIT@Work (2010) [119]	Star Topology (Bluetooth)	Adhoc, Cellular	Cellular	Healthcare, Emergency Rescue
	ALARP (2010) [120]	Wired (Mobile Terminal)	N/A	WiFi, XBee, Cellular (Base Station)	Railway Automatic Track Warning System
	CORMORAN (2011) [121]	IR-UWB Mesh	IR-UWB / Zigbee under study	IR-UWB / Zigbee under study	Navigation and Motion Capture
	CAALYX-MV (2011) [122]	N/A (Unobtrusive)	N/A	Internet	Unobtrusive Assisted Living System for Elders
	Help4Mood (2011) [123]	N/A (Unobtrusive)	N/A	Internet	Treatment of People at Home
	suWBAN (2012) [124]	Star Topology (Zigbee, Bluetooth)	N/A	Solar powered WiFi access points	Health Monitoring

**Table 14.** WBAN Design Challenges.

**Comparison Criteria**	**WBANs Solutions**	

	**General-Purpose or Medical**	**Life and Safety Critical**
Deployment Environment	Controlled	Harsh and/or unhealthy
Operation Conditions	Controlled	Presence of hazards and uncertainties
Dependence on Existing Infrastructures	High	Low
Support for Intra-WBANs	High	High
Support for Inter-WBANs	N/A	High
Support for Beyond-WBANs	High	Low to Medium
Coexistence	Mostly non-collaborative	Hybrid (collaborative, non-collaborative)

**Table 15.** Monitoring requirements for workers safety scenario.

**Categories**	**Monitoring**	**Sensors**	**Other Devices**
Health	Vital signs: heartbeat, temperature, oxygen saturation, *etc*.	Pulse count,heart rate, respiratory count, temperature, SpO_2_, ECG, EEG	Coordinator, Smart Phone
	Specialized Signals: ECG, EEG, EMG, *etc*.		

Movement & Activity	Speed and direction	Accelerometer, gyroscope, etc.	GPS

Safety and Protection	Fall detection, unusual activity detection, toxic gasses and hazards	Accelerometer, gyroscope, gas sensor	RFID, BLE

**Table 16.** Sensors, their locations and corresponding mobility for various body postures. R and L in the superscript (of sensor location) represent right and left.

**Body Position**	**Sensors**	**Sensors Location**	**Mobility**
Sitting	Heart Beat	Wrist ^R^	Low Mobility OR Static
	Temperature	Wrist ^L^	Low Mobility OR Static
	ECG	Chest	Static
	EEG	Head	Low Mobility OR Static
	Oxygen Saturation	Shoulder	Static
	Accelerometer	Leg ^R^ and arm ^L^	Low Mobility
	Gyroscope	Leg ^R^ and arm ^L^	Low Mobility

Standing	Heart Beat	Wrist ^R^	Low Mobility OR Static
	Temperature	Wrist ^L^	Low Mobility OR Static
	ECG	Chest	Static
	EEG	Head	Static
	Oxygen Saturation	Shoulder	Static
	Accelerometer	Leg ^R^ and arm ^L^	Low Mobility
	Gyroscope	Leg ^R^ and arm ^L^	Low Mobility

Walking	Heart Beat	Wrist ^R^	Low-to-High Mobility
	Temperature	Wrist ^L^	Low-to-High Mobility
	ECG	Chest	Low Mobility
	EEG	Head	Low Mobility
	Oxygen Saturation	Shoulder	Low Mobility
	Accelerometer	Leg ^R^ and arm ^L^	Low-to-High Mobility
	Gyroscope	Leg ^R^ and arm ^L^	Low-to-High Mobility

Running	Heart Beat	Wrist ^R^	High Mobility
	Temperature	Wrist ^L^	High Mobility
	ECG	Chest	Low-to-High Mobility
	EEG	Head	Low-to-High Mobility
	Oxygen Saturation	Shoulder	Low-to-High Mobility
	Accelerometer	Leg ^R^ and arm ^L^	High Mobility
	Gyroscope	Leg ^R^ and arm ^L^	High Mobility
